# The hazards of smoking and the benefits of cessation: A critical summation of the epidemiological evidence in high-income countries

**DOI:** 10.7554/eLife.49979

**Published:** 2020-03-24

**Authors:** Prabhat Jha

**Affiliations:** Centre for Global Health Research, Dalla Lana School of Public Health and Unity Health, Toronto, University of TorontoOntarioCanada; McGill UniversityCanada; McGill UniversityCanada

**Keywords:** tobacco, hazards, high-income countries, smoking, mortality

## Abstract

In high-income countries, the biggest cause of premature death, defined as death before 70 years, is smoking of manufactured cigarettes. Smoking-related disease was responsible for about 41 million deaths in the United States, United Kingdom and Canada, cumulatively, from 1960 to 2020. Every million cigarettes smoked leads to one death in the US and Canada, but slightly more than one death in the UK. The 21^st^ century hazards reveal that smokers who start smoking early in adult life and do not quit lose a decade of life expectancy versus non-smokers. Cessation, particularly before age 40 years, yields large reductions in mortality risk. Up to two-thirds of deaths among smokers are avoidable at non-smoking death rates, and former smokers have about only a quarter of the excess risk of death compared to current smokers. The gap between scientific and popular understanding of smoking hazards is surprisingly large.

## Overview

I summarize the causative role of smoking for the most common causes of death among adults in high-income countries, drawing on data from Canada, the United States (US) and the United Kingdom (UK). The main objective of this analysis is to review the hazards of smoking and the benefits of cessation. I do so by examining the cause, nature and extent of tobacco-related diseases in high-income countries between 1960 and 2020. The review has seven main conclusions.

First, in much of Europe and North America, the biggest cause of premature death, defined as death before 70 years, is smoking of manufactured cigarettes. Smoking as an important cause of many diseases in many populations has been recognized widely in the scientific literature for the last five decades. However, three surprising features of health hazards of smoking have been established reliably only since about 2012. The first feature is that risk of developing disease among smokers is big. The second feature is that for smokers to develop these big risks, they need to start smoking early in adult life and to continue smoking. If smokers don’t start early in life, their risks are substantially smaller. Third, if smokers stop smoking before they develop some serious disease, then their risks are substantially reduced.

However, most smokers whom start early in adult life and who continue to smoke are eventually killed by their tobacco use. This is because in every year during middle age (defined here as ages 30–69 years), the death rates among smokers are about three-fold higher than that of similar non-smokers (considering differences between smokers and non-smokers in heavy alcohol use, obesity patterns or different educational or economic status). So two-thirds of the mortality among smokers would not be happening if they had the non-smoker death rates. Most of this excess risk arises from diseases that are caused by smoking. This includes disease such as lung cancer, emphysema, heart attack, stroke, cancer of the upper aerodigestive areas, bladder cancer and various other conditions. Thus this excess risk of disease and death is a cause and effect relationship.

Second, despite substantial declines in the proportion of adults who smoke in most high-income countries, cigarette smoking remains a common exposure in many countries. There were approximately 34 million smokers in the US, 7 million in the UK and 5 million in Canada in 2017 and the number of cigarettes sold in recent years has remained mostly unchanged for the past decade in Canada, while it has declined in the US and the UK. In recent years, electronic cigarettes appear to have accelerated the decline in smoking among younger adults. E-cigarettes are far less hazardous than cigarettes, but do carry some risks, most notably the risk of addiction to nicotine among youths.

Third, a proper understanding of the hazards of smoking requires due consideration of the lag of decades between onset of smoking and the development of disease(s). For both individuals and populations to experience increases in the risk of death, prolonged smoking from early adulthood without cessation is required. The increases in the risk of death can be gauged reliably by studying trends in national lung cancer mortality of different generations. The age-specific health hazards can also be documented in large prospective studies, which monitor groups of smokers and non-smokers for the development of disease(s) over time. Conservatively, smoking-related disease was responsible for about 41 million deaths in the US, UK and Canada, cumulatively, from 1960 to 2020. Every million cigarettes smoked causes approximately one death in the US and Canada, but about 1.3 deaths in the UK.

Fourth, the hazards of smoking are much bigger than was documented just two decades ago. Differences in death rates among smokers and non-smokers imply that smokers lose on average at least a decade of life. About half of all smoking-attributable deaths occur in middle age. The specific conditions caused by smoking include vascular, respiratory and neoplastic (cancer) disease (which account for approximately 75% of all causes of death in most high-income countries). Smoking is increasingly linked to conditions such as diabetes, rheumatoid arthritis, age-related macular degeneration of the eye, orofacial clefts and ectopic pregnancy. Indeed, the list of smoking-attributable diseases continues to expand with additional studies and monitoring. Hence, total mortality differences between otherwise similar smokers and non-smokers are a robust yet simple way to estimate the effects of smoking.

Fifth, cessation is effective in reducing the increased risks of developing smoking-related disease. Smokers who successfully quit before age 40 avoid nearly all increased mortality risks of continued smoking. Smoking cessation yields specific benefits of reducing fatal and non-fatal vascular, respiratory and neoplastic disease. Former smokers have about only a quarter of the excess risk of death than do current smokers. Studying cessation provides further evidence about the causal nature of smoking and disease development.

Sixth, the biological evidence about particular carcinogens and other toxins found in manufactured cigarettes and the possible mechanisms that trigger heart attacks and strokes are consistent with the epidemiological evidence. Genetics is an important factor in disease susceptibility but does not negate the substantial importance of smoking in explaining the marked changes in cause-specific mortality and total mortality attributable to smoking over the last few decades.

Finally, there continues to be gross underestimation of the health hazards of smoking by the public, non-experts and even some experts. The large health risks inherent in smoking are often wrongly equated with the far smaller risks of other health exposures. For example, smoking remains far more hazardous to the individual adult in high-income countries versus moderate obesity, heavy alcohol use and other factors. Most adults surveyed in the US remained surprisingly unaware of the high levels of disease risk that occur today from smoking.

## Introduction

Tobacco use is well established as a major cause of death worldwide, accounting for about five to six million deaths per year worldwide (Jha and Peto, 2014). On current smoking patterns, about one billion deaths may occur from smoking during the 21^st^ century, in contrast to ‘only’ 100 million deaths in the 20^th^ century (Peto et al., 1994). Already about 100 million tobacco deaths have occurred this century and there will be another 250 million tobacco deaths before 2050. The vast majority of the deaths before 2050 will occur among current smokers. Hence, the major public health priority is to increase the proportions of adults who quit smoking as well as to reduce the uptake of smoking by young adults and children.

In order for individuals to properly understand their risks of smoking, an understanding of the risks of smoking and benefits of cessation is required. This in turn requires a detailed understanding of the cause, nature, and extent of tobacco-related disease, including an understanding of the evolution of cigarette smoking in populations. Relationships between smoking and disease depend on changes in consumption patterns of smokers and ingredients of commonly manufactured cigarettes. This review is intended to inform governments, researchers, health care providers and individuals about the contemporary hazards of smoking. To do so, I outline the contemporary evidence that defines the causal relationship between smoking and the development of major causes of premature death. I focus mostly on evidence relevant from the US, UK and Canada, as typical of most high-income countries.

As most of the evidence regarding the relationship between smoking and disease has been on mortality, I focus on mortality by cause. Mortality has less misclassification than non-fatal outcomes, such as a first or recurrent non-fatal heart attack (Jha, 2014; Menon et al., 2019). However, I assess to some extent the evidence on the development of new diseases in previously non-diseased (or healthy) populations (or incidence).

This review is in eight sections. This first section covers key sources of data and methods. Section two reviews the current number of smokers in the US, UK and Canada. It also looks at historical trends in smoking, specifically trends from 1920 to 2010, with an emphasis on trends over the last five decades. It describes changes in the manufactured cigarette which have influenced the addictive properties of cigarettes, and thus, the risks of disease development. A brief review of electronic cigarettes follows.

Section three focuses on smoking as a cause of disease. It reviews, briefly, the history of studies linking smoking to disease, with particular attention to the importance of prolonged smoking to the development of disease. Because lung cancer is a highly-studied disease, this section draws upon national rates of lung cancer mortality and prospective studies of individuals who were smokers or non-smokers and who were observed for the development of the disease. This section also provides estimates of total mortality from smoking from 1960 to 2020 in the three countries (totalling about 41 million deaths), including the relationship of number of cigarettes smoked with mortality.

Section four reviews the epidemiological evidence that relates smoking to both total mortality and cause-specific death rates from the most established conditions linked to smoking. It provides estimates of the avoidable proportion of deaths had the smokers had death rates of comparable non-smokers. Section five outlines the recent evidence on the reduction in total mortality and cause-specific mortality from smoking cessation. Cessation is a powerful way to establish the causal relationships between smoking, total mortality and specific diseases. This section also quantifies the reduction in excess risk among former smokers compared to continued smoking.

Section six briefly summarizes the biological evidence regarding the link between nicotine addiction and smoking, and between smoking and disease. It outlines how biological data can help dispel some commonly held myths about the ‘genetic basis’ of smoking-attributable disease. This summary reemphasizes the central point that reliable quantification of smoking hazards is best done at the level of populations, using epidemiological studies to determine both health risks in individual humans and death rates in populations. Section seven reviews and contrasts the commonly perceived risks of alcohol use and pollution or environmental exposures that influence human health to epidemiological findings on hazards of smoking. Section eight discusses implications for future research and provides a brief conclusion.

This review focuses on the *consequences* of smoking, and not on the *causes* of smoking (including the key biological, social, economic and marketing influences that lead to variable rates of smoking initiation and cessation across different populations). However, I do examine the engineering of addictive nicotine to the modern manufactured cigarette, which plays a central role in explaining smoking patterns. For a careful review of the causes of smoking, I refer the reader to various US Surgeon General Reports (USSGR), most notably those of 1989 and 2014 (U.S. Department of Health and Human Services, 1989; U.S. Department of Health and Human Services, 2014). Similarly, the emphasis is on high-income countries and not low and middle-income countries, where the evolution of the tobacco epidemic is not yet mature, and which has much lower rates of cessation (Jha and Peto, 2014). The issues related to disease patterns in low and middle-income countries are also quite distinct (Jha and Peto, 2014; International Agency for Research on Cancer (IARC), 2004; Jha et al., 2008; Liu et al., 1998). The review of the consequences of smoking does not include second-hand smoking. Rigorous assessments on the relationship of second-hand smoking to cancer and other diseases have been completed by the International Agency for Research on Cancer (IARC Working Group on the Evaluation of Carcinogenic Risk to Humans, 2004; International Agency for Research on Cancer (IARC), 2012), and the US Surgeon General (USSGR; U.S. Department of Health and Human Services, 2004; U.S. Department of Health and Human Services, 2014). Finally, this does not focus on *control* of smoking, including the most important role of higher exicse taxes to raise cigarette prices, for which there are several reviews and WHO reports (Jha and Chaloupka, 1999; Jha et al., 2015; World Health Organization, 2017; Jha and Peto, 2014).

### Sources of evidence

The main sources of evidence in this report are the published literature, which is accessible publicly through the PubMed portal (https://www.ncbi.nlm.nih.gov/pubmed/); scholarly summations done by key technical agencies such as the US Surgeon General, International Agency for Research on Cancer (IARC), WHO, the US Institute of Medicine, and other independent technical groups; and my own epidemiological research. Like most academic researchers, I did not have access to the scientific research conducted by tobacco industries, much of which remains closed to the public.

The office of the US Surgeon General periodically assembles global evidence on the hazards of smoking (https://www.surgeongeneral.gov/library/reports/). The most important of these compiled evidences was the 1989 report covering the 25 years of progress after 1964 (when the first US Surgeon General’s report on smoking was published) and the 2014 report covering 50 years of progress (U.S. Department of Health and Human Services, 1989; U.S. Department of Health and Human Services, 2014). IARC publishes similarly comprehensive reviews of known carcinogens in humans and has examined tobacco in these reviews over various years (http://monographs.iarc.fr/), with the most notable being the 2004 report (IARC Working Group on the Evaluation of Carcinogenic Risk to Humans, 2004). Finally, in 1981, an influential report for the US Congress Office of Technology Assessment concluded that tobacco smoking was the leading cause of cancer deaths in the US, accounting for more avoidable deaths than the sum of several pollutants or other environmental exposures ( Doll and Peto, 1981).

The authors of these aforementioned reviews have followed reasonably strict rules to assess evidence. These rules require that in considering the result of any particular study, reviewers are to examine if bias, confounding or misclassification of exposure or outcome could explain the observed results. Only those studies that examines such biases, and ensure that such biases do not account for the observed relationship of smoking and disease are included into any quantitative synthesis of the evidence. Many of the sources of data in this report are from the US and the UK, where there have been more studies over longer durations done than in Canada or other high-income countries. There are, of course, some differences between American,Canadian, and UK citizens and populations, in terms of disease distribution, access to health-care and other factors, as well as in the ingredient formulation of the most common cigarettes smoked. However, these differences are quite small compared to the similarity of mortality rates from specific causes, such as particular cancers, vascular and respiratory diseases (World Health Organization, 2016).

The three countries have similar rates of exposure to smoking (beginning in early adulthood), among a substantial proportion of adults. The differences in disease risks between smokers and non-smokers due to prolonged smoking are very large for many diseases. Hence, differences in disease patterns or smoking product do little to alter the main conclusions about the degree to which smoking is a causative factor for the development of the diseases common in most high-income countries, including those outside Canada, the US and the UK.

A central consideration of this epidemiological evidence is the delay between the onset of smoking in adolescence or early adult life and the development of disease in middle-age, implying a lag between initial exposure and eventual effect. This delay between the onset of smoking and its potential eventual consequences is a major source of confusion among the public, non-experts, and even some experts, about the causal relationship between smoking and specific diseases. Hence, I pay detailed attention to describing the full risks where they are already documented and point out populations (such as women) and diseases for which such risks are still not fully documented.

## Smoking trends in the US, UK and Canada

Despite substantial declines in smoking, a large proportion of Americans, Canadians and UK citizens continue to smoke. Largely attributable to the addictive nature of cigarettes, the declines in smoking prevalence have not been matched by declines in the number of cigarettes smoked daily by continuing smokers.

### Current smoking prevalence

In the US in 2017, an estimated 34.3 million adults aged 18 and older or 14.0% of US adults smoke cigarettes (Centers for Disease Control and Prevention, 2018). The smoking prevalence is higher among men than women (15.8% vs 12.2%). In the same year, 7.4 million adults in the UK smoked, or 15.1% of adults (17.0% of men and 13.3% of women) (Office for National Statistics, 2018a). In Canada, about 5 million Canadians smoked (16.2% of those aged 12 and older; 19.1% of men and 13.4% of women [Statistics Canada, 2019]). The majority of the current smokers (meaning those those who report themselves as non-daily or daily smokers) smoke daily. Other types of tobacco use are less common than cigarettes, with cigars and cigarillos smoking reported by 4% of Americans and 3% of Canadians (Centers for Disease Control and Prevention, 2018; Reid et al., 2017).

Current trends in smoking prevalence reflect a combination of those that smoke daily or occasionally, former smokers and people who never started smoking. The prevalence of smoking is also affected by changes in the denominators of all people, including immigration, which tends to lower smoking prevalence (Newbold and Neligan, 2012). I examine historical trends in smoking by sex. This is significant as women’s smoking has increased substantially the over the last few decades.

### Historical smoking trends and changes in prevalence in the last four decades

Prior to 1900, most tobacco consumed was in the form of chewed tobacco, snuff, pipes and cigars. Some of the first reports of smoked tobacco came from Spain, where beggars in the 16^th^ century collected discarded cigar butts, shredded them and rolled them in scraps of paper for smoking. These ‘poor man’s cigars’ were known as *cigarillos* (which translates, from Spanish, to ‘little cigars’). Late in the 18th century, cigarillos acquired respectability. Their use spread throughout Europe, aided by their popularity among troops in the Napoleonic Wars. The French named them cigarettes. British tastes switched to cigarettes filled with unmixed Virginia tobacco, while the US market preferred blended tobacco (Encyclopaedia Britannica, 2018).

Prior to the early 20th century, cigarettes were made by hand, either by the smoker or in factories. The factory process consisted of hand-rolling on a table, pasting and hand-packaging. In 1880, American James A. Bonsack was granted a US patent for a cigarette machine. Automated machines could produce 120,000 cigarettes in ten hours (approximately 200 per minute). This machine revolutionized the industry and supported a substantial expansion of the major cigarette companies in the US, as well as exports to the UK and European countries. By 1920, there was a marked increase in the use of cigarettes in much of Europe and North America and accelerated further during World War II (WWII), when cigarettes were part of soldier rations. This dissemination contributed to a major expansion in cigarette smoking during the first-half of the 20th century, displacing pipe smoking, chewed tobacco, snuff, cigars and other types of tobacco product (Encyclopaedia Britannica, 2018; Thun et al., 2002).

**Figure 1.**
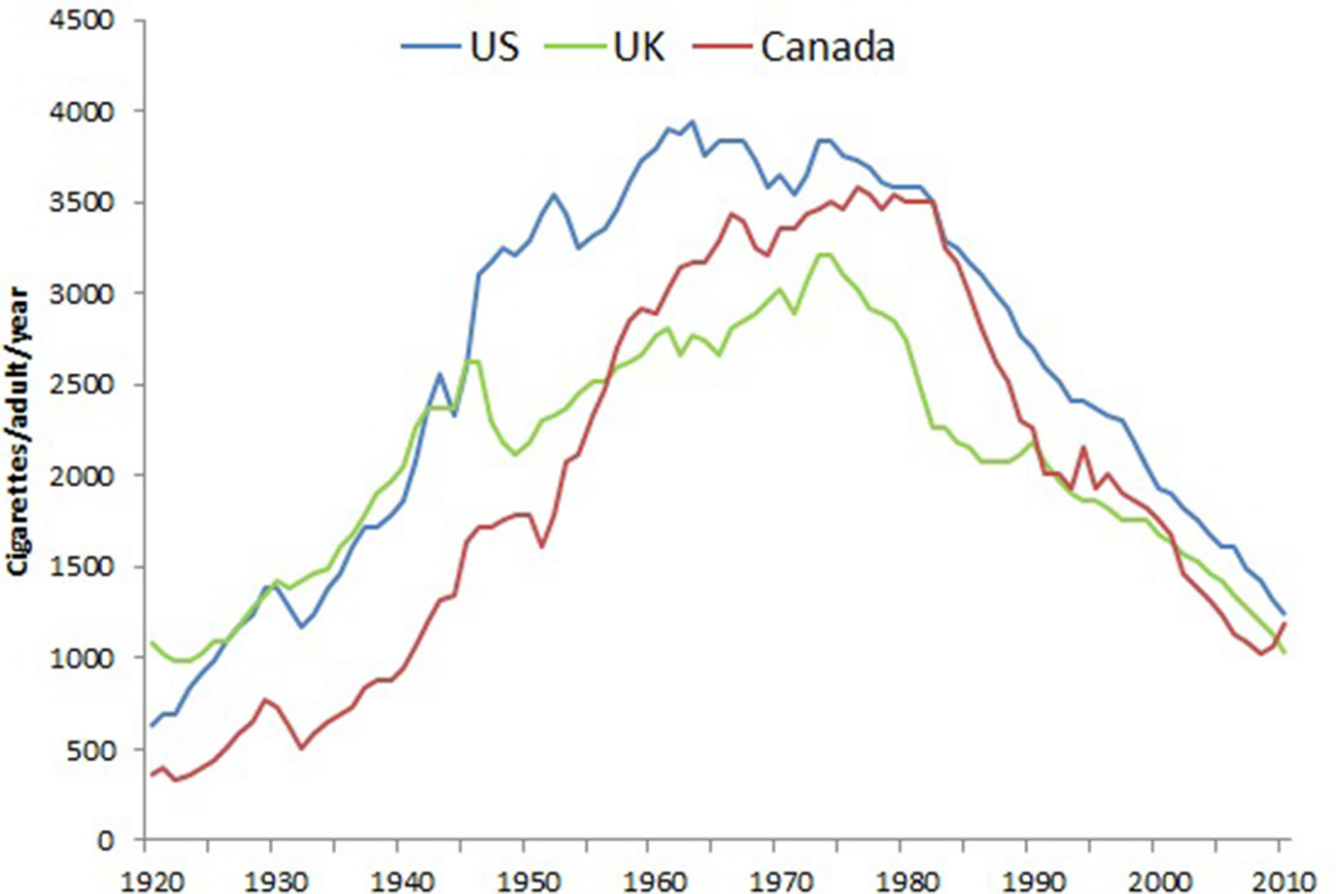
Trends in per capita consumption in Canada, US, UK in cigarettes per adult per year. Note. Data from Forey et al. (2016), *International smoking statistics.*

Figure 1 provides the per capita cigarette consumption in the US, Canada, and the UK from 1920 to 2010, based on a global compilation of sales data that includes tobacco industry sources (Forey et al., 2016). Sales data provide a useful indicator of overall consumption trends over prolonged periods. These data are obviously crude and subject to various reporting errors, such as illegal or undocumented sales, which in recent years has paralleled the increase in smuggling; given the tobacco industry’s active role in smuggling their own products (Merriman, 2012). Furthermore, sales data do not capture changes in the length of manufactured cigarettes, the amount of tobacco within various lengths, and mostly do not capture gender-specific smoking patterns, including the notable time lag between increased male smoking and female smoking in the three countries. Importantly, peak consumption among American males, as reported from prevalence surveys, was the year 1963, when overall US cigarette sales also peaked. In contrast, in the UK and in Canada, peak sales occurred around 1975 to 1980.

**Figure 2.**
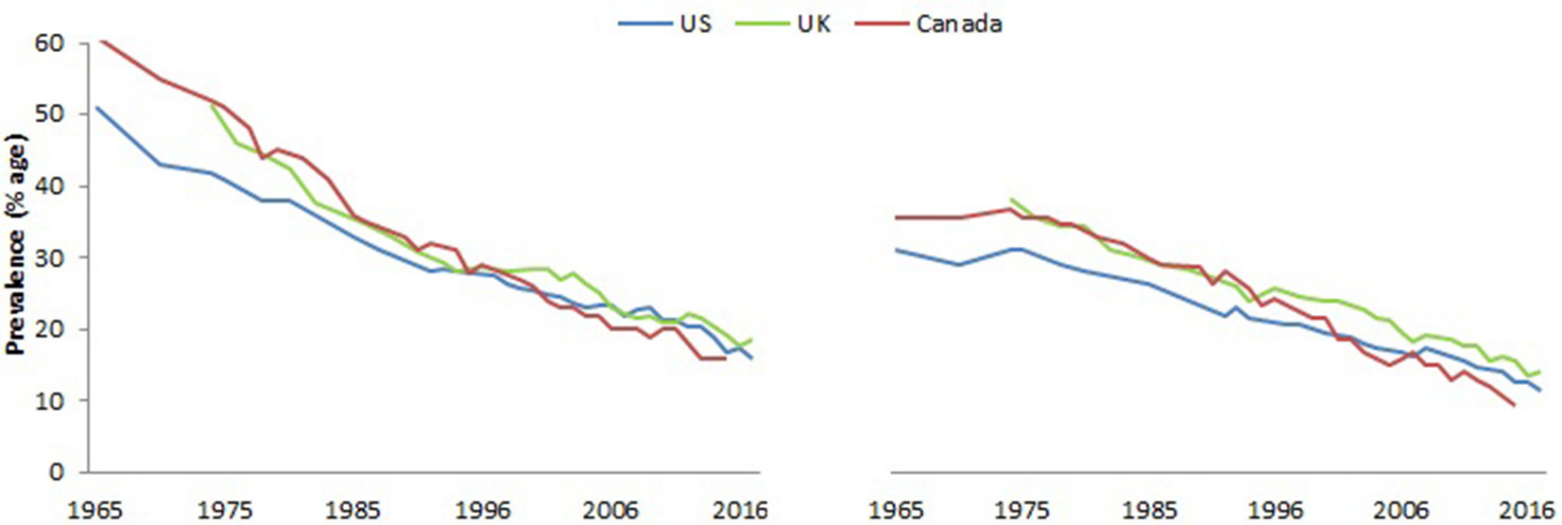
Prevalence (%) of adult males (right panel), and females (left panel), usually 15 to 18 years or older that smoke by sex in US, UK and Canada. Note. Data from National Center for Health Statistics, 2008, 1965–2012 National Health Interview Survey (NHIS); Office for National Statistics, 2018, *Adult Smoking habits in Great Britain*; and Reid et al., 2017, *Tobacco Use in Canada: Patterns and Trends, 2017 Edition*.

Figure 2 shows trends in overall smoking prevalence in both sexes over the last five decades in the three countries, during which better-quality surveys of smoking prevalence in the adult population became available. These surveys document the steady decline in smoking prevalence that began around the same time as the publishing of the 1962 Royal College Report in the UK and the 1964 Surgeon General’s report in the US, both which provided expert opinion linking smoking to lung cancer (Royal College of Physicians, 1962; U.S. Department of Health, Education, and Welfare, 1964).

In most high-income countries, there has been a notable reduction in heavy smoking (over 20 cigarettes/day). For example, in the UK, in 1974, 26% of men and 13% of women were heavy smokers compared to 5% of men and 3% of women in 2012.

The average number of cigarettes smoked per day by men and women has decreased across all age groups, consistent with the declines of per capita consumption as shown in Figure 2 (Action on Smoking and Health, 2016). Nevertheless, significant amounts of smoking defined as at least half a pack (ten cigarettes) a day persists in many populations.

From 2000 to 2015, smoking prevalence fell steadily by well over a third in the US, UK and Canada (Table 1). By contrast, the reduction in smoking amount among daily smokers has been more modest, at about one-fifth.

**Table 1.** Adult smoking prevalence (daily and non-daily) and daily cigarettes for US, UK, and Canada in 2000 and 2015.

Year	2000	2015	Change in %
Prevalence*			
US	23.1	15.2	−34.2%
UK	27.0	17.8	−34.1%
Canada	24.4	13.0	−46.7%
**Amount per day (among daily smokers)**			
US	18.1	14.2	−21.5%
UK*	13.8	11.3	−18.1%
Canada	16.8	13.8	−17.9%

^*^Daily and non-daily smokers. *Note.* Data from National Centre for Health Statistics, *NHIS;* Office of National Statistics, *Adult Smoking habits in Great Britain*; Statistics Canada and Reid et al., 2017, *Tobacco Use in Canada: Patterns and Trends, 2017 Edition* –Defined as having smoked in the past 30 days and having smoked 100 cigarettes in a lifetime. Adult ages are 15+ in Canada and the UK and 18+ in the US.

The main conclusion remains that in these three countries, about one in six to one in seven of adults, were smokers in 2017. In absolute terms, this represents about 45 million cigarette smokers in the three countries.

### Changes in the manufactured cigarette

Here I will outline three documented strategies used by the tobacco industry to increase uptake and maintain behaviour of smoking (construction, tar content, and use of filters). The tobacco industry conducts much of the scientific research on nicotine, addiction and the role of advertising and promotion to start and maintain tobacco addiction. Little of this research is available publicly.

Thun et al. (2002) point out that the change in the manufacturing process in the US starting about 1930 for cigarettes resulted in increased exposure to surfaces within the respiratory tract. Snuff and other smokeless tobacco, much of which was commonly used before 1900, exposed the local areas of the lip and the oral cavity, as well as extracts absorbed in saliva, and hence caused mostly oral cancers. The smoke from cigars, pipes and traditional roll-your-own cigarettes was traditionally strongly alkaline, which discouraged deep inhalation. Early cigarettes released an un-ionized form of nicotine that could be absorbed by the linking of the mouth and upper airway. Improvements in cultivation and processing lowered the acid content of cigarettes and made them easier to inhale. These improvements also allowed for the release of ionized nicotine, which could be absorbed by the lower parts of the lung (including the tracheal and large bronchi). Thus, changes to the manufactured cigarette shifted the location of cancers from the upper airways to those of the trachea, bronchus and lung.

Reported levels of tar content in manufactured cigarettes have dropped substantially over time. However, a large UK study showed that even low-tar cigarettes sharply increased rates of myocardial infraction. Especially among smokers in their Thirties, Fourties or Fiftees much of the excess risks of continued smoking are avoided by cessation, and less so by changing from one type of cigarette to another (Parish et al., 1995).

Most, but not all, reviews of changes in tar content in US cigarettes have concluded that while there might be some reductions in lung cancer from smoking lower-tar cigarettes, the overall risks of disease are not greatly diminished (Thun and Burns, 2001). The Institute of Medicine (Bondurant et al., 2001), and the National Institutes of Health, National Cancer Institute, 1996, have examined evidence that low-tar-yield cigarettes reduce disease risk and concluded, “*there have been many efforts in the past to develop less harmful cigarettes, none of which has proved to be successful.”* The UK Royal College of Physicians (2000) reached similar conclusions. The National Cancer Institute review concluded: “*Epidemiological and other scientific evidence, including patterns of mortality from smoking caused diseases, does not indicate a benefit to public health from changes in cigarette design and manufacturing over the last 50 years*.” Lower-tar cigarettes do appear to result in lower lung cancer deaths. However, there might well be compensatory smoking among smokers who smoke these lower-tar cigarettes, in which the smoke inhalation tends to be more forceful, pulling the smoke deeper into the lungs (Thun and Burns, 2001).

Filters have been in place in most cigarettes for the last four decades. The purpose of the filter is to reduce the amount of tar, smoke, and fine particles inhaled from combustible tobacco, as well as to reduce tobacco flakes from entering the mouth. Many filters are perforated with small holes that intend to dilute the inhaled smoke with external air. When machines are used to test these cigarettes, the findings tend to assign the content of these cigarettes as low-tar or low-nicotine. However, smokers cover these ventilation holes with their lips or fingers. There is also evidence that smokers inhale filtered cigarettes more deeply. The combination of these factors means that these so-called ‘safer’ cigarettes are, in fact, no safer than others (Kozlowski et al., 1998).

In conclusion, smoking is best understood largely as a manifestation of nicotine addiction. The design of the Western, manufactured cigarette likely considers the optimization of initiation and addiction maintenance (U.S. Department of Health and Human Services, 1990). Additional social cues, achieved by mass-marketing, and policies that enable smoking in social setting, such as bars and restaurants, might well enforce the addictive properties of smoking, increasing uptake rates and making cessation less common (U.S. Department of Health and Human Services, 2010a; U.S. Department of Health and Human Services, 2020).

### Recent emergence of e-cigarettes

Alternative nicotine delivery systems include lower-risk nicotine and tobacco products like nicotine replacement therapy pharmaceuticals, low-nitrosamine smokeless tobacco products, and most notably electronic -cigarettes (also referred to as ‘vaping’ products). E-cigarettes were introduced around 2010 but have become particularly popular since about 2015, as they mimic the look and feel of conventional cigarettes (unlike nicotine chewing gum or patches). They are hand-held, generate a smoke-like vapour and hence recreate sensations similar to smoking the smoke from conventional cigarettes. E-cigarettes can be used with or without nicotine. Much of the recent attention in the US has been on the ‘JUUL’ (which has high doses of nicotine) and in Japan on the ‘iQOS’ product (which heats tobacco to generate a nicotine vapour but does not burn it) (Foundation for a Smoke-Free World, 2018; U.S. Department of Health and Human Services, 2016). There has notable increase in e-cigarette use in many high-income countries, particularly among youth (Thatcher, 2015). A full review of e-cigarettes is beyond the scope of this review, but Warner (2019) provides useful suggestions for regulation based on the limited evidence base.

E-cigarettes have lower levels of possible carcinogens and toxins than conventional cigarettes, such as 450-fold and 120-fold lower levels of acetaldehyde and toluene, respectively (Goniewicz et al., 2014). E-cigarettes are not completely risk free, as they contain nicotine which has short-term cardiac and other effects. The recent reports of lung-injury among e-cigarette users appears to mostly arise from tampering with products to add marijuana and other agents, and not from the nicotine or flavoring of most e-cigarettes (Blount et al., 2020). Moreover, in contrast to cigarettes, long-term studies of e-cigarettes use to determine the mortality risks among users and quitters have not yet been completed. Nonetheless, there is general consensus that e-cigarettes are considerably safer than cigarettes. The National Academies of Sciences, Engineering, and Medicine, 2018 report noted “*conclusive evidence that completely substituting e-cigarettes for combustible tobacco cigarettes reduces users’ exposure to numerous toxicants and carcinogens present in combustible tobacco cigarettes”* and “*substantial evidence that completely switching from regular use of combustible tobacco cigarettes to e-cigarettes results in reduced short-term adverse health outcomes in several organ systems.”*

The use e-cigarettes increased most sharply in the US and Japan from about 2015 onwards, and has led to a major major debate if e-cigarettes act as a ‘gateway’, to encourage youth to smoke cigarettes who would not otherwise take up smoking. There have been competing, limited and generally, short-term studies, and these have methodical challenges in determining if the kids who take up e-cigarettes are the ones who might have smoked cigarettes anyway. Moreover, limitations about the sample size, use of many types of vaping products (including some without nicotine), and duration of follow up that limit definitive conclusions (Warner, 2019).

However, a few key features of the trends in use of e-cigarettes by youth are already clear. First, even prior to widespread use of e-cigarettes, prevalence and initiation rates of cigarette smoking were falling in youth in the US, UK and Canada. E-cigarette use seems to have accelerated declines in cigarette smoking, particularly for the age group cohorts most likely to use e-cigarettes. For example, from 2010 to 2017, in the US and the UK overall cigarette prevalence fell by an absolute 5% (from 19.3% to 14.0% in the US and 20.1% to 15.1% in UK). At ages 18–24 years, prevalence in the US by 10% (from 20.1% to 10.4%) and in the UK it fell by 8% (from 25.5% to 17.8%) (Office for National Statistics, 2018b; Centers for Disease Control and Prevention, 2019).

Across the three countries, most e-cigarette experimentation does not appear to turn into regular use. In the Canadian and UK studies, the youngest age groups appear to have a greater proportion of e-cigarette among never smokers than at older ages, perhaps reflecting a shift to vaping alone. Among adolescents, most of the uptake of e-cigarettes has been among current or past cigarette smokers. In the US, among high school students (15–18 years), only 0.3% were frequent e-cigarette users (20 or more days of past 30), and 4.6% used any in the past 30 days. As compared to never smokers, cigarette smokers were much more likely to vape daily (16.9% v 0.2%), or frequently (21.8% v 0.3%) (Levy et al., 2019) In the UK, among 11–18 year old vapers, only 0.8% were among never smokers, versus 40.3% of smokers and 12.2% of former smokers (Action on Smoking and Health, 2019). Finally, in Canada, 83% of vapers were current (60%) or former smokers (23%) with only 16% of vapers among never smokers (but more in age group 15–19 years; Environics Research, 2019).

There is little evidence to justify the claim that e-cigarettes leads to increases in cigarette smoking by youth in the US (Warner, 2019), or the UK (Bauld et al., 2017). Notably, the reported patterns show that daily e-cigarettes use (typically over the last 30 days) remains generally lower than for cigarettes (Levy et al., 2019) suggesting that while youth might be experimenting, e-cigarette is not fully displacing cigarette use. Moreover, in the US, it appears that even if increased e-cigarette use by never-smoking adolescents raises their risk of trying smoking, that effect is more than offset by the longer secular trends of falling cigarette smoking (Warner, 2019).

The major increase in vaping in youth has not occurred in the UK, perhaps because regulations cap maximal nicotine content at <20 mg/ml (which are the European Union caps for cigarettes). By contrast, the current Canadian limits are 65 mg/ml and the JUUL product in the US is widely sold at 59 mg/ml. Moreover, the UK has taken mostly a harm reduction strategy toward e-cigarettes, including regulations on marketing and promotion. The US and some Canadian provinces, such as Ontario, have little regulation, and substantial social media campaigns try to increase uptake by young adults (U.S. Department of Health and Human Services, 2020; Cummings and Hammond, 2020). Finally, there are differences across the three countries in marketing efforts, regulations, and in the effects of peer-influences in US high schools.

The most important motivator for adults in several high-income counties to use e-cigarettes has been to decrease the amount smoked or to quit (Riahi et al., 2019). A Cochrane Collaboration review concluded that based on three randomized trials, that e-cigarettes containing nicotine did help smokers stop smoking in the long term compared with placebo e-cigarettes (Hartmann-Boyce et al., 2016). A more recent randomized trial in the UK found that e-cigarettes achieved about twice the cessation rates at one year than did users of nicotine patches (which are well established to aid cessation; Hajek et al., 2019). Finally, the population-level impact of e-cigarettes on adult cessation in the US and UK suggests that their introduction has accelerated adult cessation rates somewhat (Zhu et al., 2017; Beard et al., 2016).

In conclusion, far more epidemiological evidence is needed to settle the ongoing heated debates about e-cigarettes. The most important question is to better understand the extent to which e-cigarettes might help the very large numbers of current smokers in the world to quit, given the overwhelming evidence on the benefits of quitting cigarettes.

## Smoking as a cause of disease

### History of studies linking smoking to disease

Lung cancer was a rare disease in most high-income countries in the 19^th^ and early 20^th^ centuries. By the early 20^th^ century, most deaths were registered and certified by doctors, and these routine mortality statistics showed a very large increase in lung cancer mortality, particularly among urban men. Several cancer registries also showed a major increase in new-lung cancer cases in men in the early 20^th^ century, for example some 15-fold increases in the UK. The reasons for this marked increase in lung cancer were believed to be from better detection and diagnosis or from car exhaust (as men were more exposed than women were). However, researchers also noted a large simultaneous increase in male smoking.

**Figure 3.**
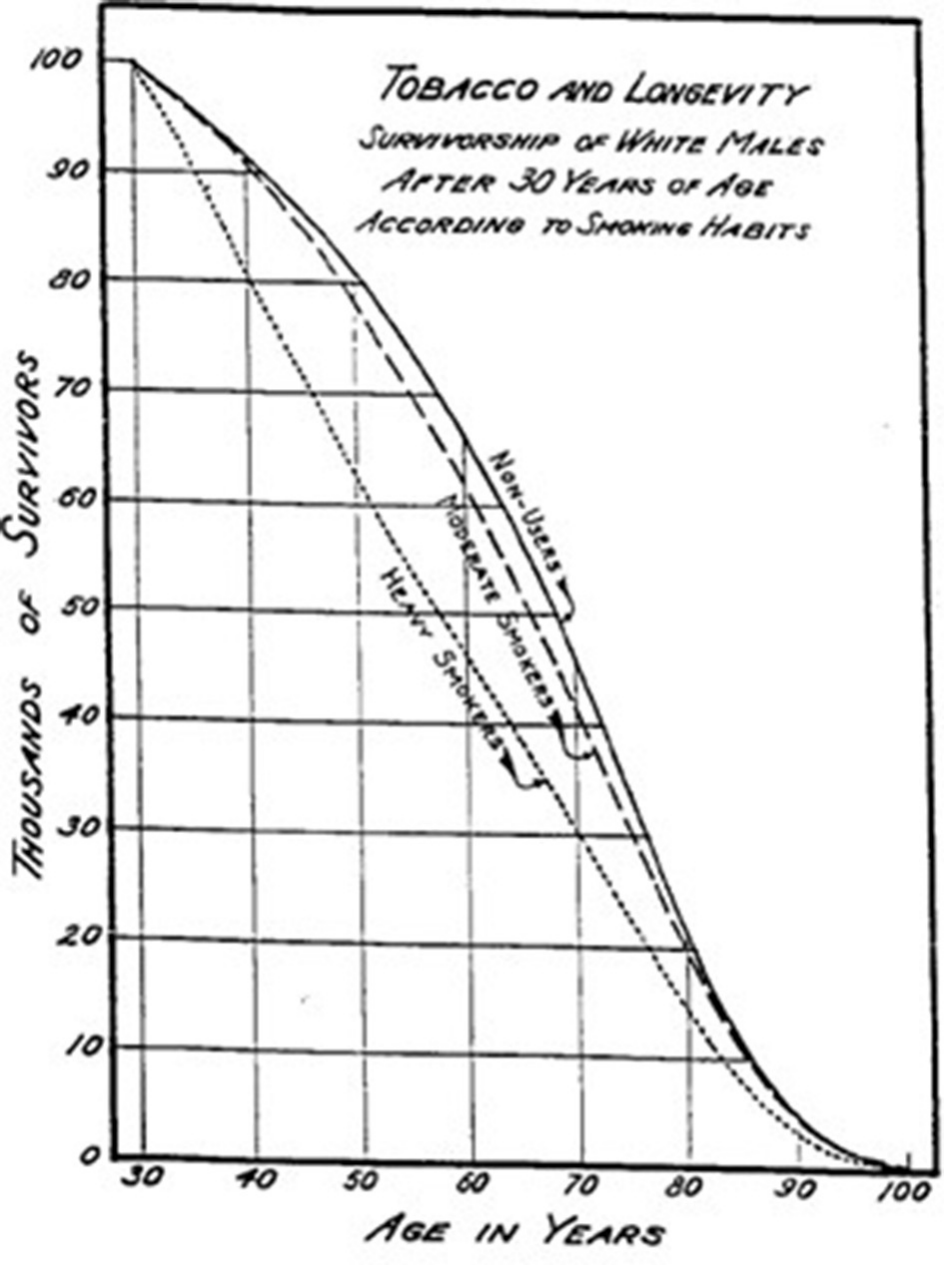
Survival by smoking among US insured adults.

By the 1930s, preliminary investigation of the parallel rise in cigarette consumption and lung cancer adopted ‘case-control’ epidemiology. Two studies published in German language in 1939 and 1943 used a ‘case-control’ methodology that examined the smoking histories of adults with lung cancer in contrast to cancer-free controls (Müller, 1940; Schairer and Schöniger, 1944). Both studies showed that most lung cancer cases smoked cigarettes. These studies were noticed within Germany (Bachinger et al., 2008), but were not widely cited in the English-language scientific literature until much later, due in part to WWII. Curiously, a US mathematician, Raymond Pearl used a different method-analysis of the insurance records of 7,000 US adults from the early 1930s to report significant (perhaps implausibly large given that smoking prevalence peaked after this time period) reductions in survival among smokers (Pearl, 1938; Figure 3).

While cited by some in the medical literature, Pearl’s finding was also largely ignored. This was, in part, because the ideas of causation in epidemiology were not well-defined, and most people dismissed these findings as chance correlations.

Major breakthroughs came with several near-simultaneous epidemiological studies published around 1950, that also used the case-control methodology, including by Ernst Wynder and Evarts Graham in the US and Richard Doll and Bradford Hill in the UK (Wynder and Graham, 1950; Doll and Hill, 1950). The Doll and Hill paper showed that cigarette smoking was far more commonly reported among patients with lung cancer than those with other diseases or those free of disease. The obvious criticism made of these studies was that of ‘recall bias;’ meaning that those with lung cancer were more likely to remember a history of smoking than those without. However, the marked differences in the prevalence of smoking between lung cancer cases and controls were far beyond that which could be expected simply from differing memories. Moreover, Doll and Hill showed that the prevalence of smoking among a subset of men who were suspected of lung cancer but were subsequently shown to have other ailments, were very similar to the control men.

Doubts persisted in the popular press and among medical establishments, due, in part, to the fact that about 80% of UK doctors themselves smoked during the 1950s. Around 1955, further epidemiological studies confirmed the striking role of smoking in development of lung cancer using a series of prospective ‘cohort’ studies, conducted to eliminate the possibility of diseased individuals remembering their smoking history more accurately than healthy individuals. Doll and Hill recruited about 40,000 doctors in the UK. This cohort was reasonably homogenous in race, social status and other factors, had strong medical record-keeping and completed questions promptly. Doll and Hill followed groups of doctors who smoked and groups who did not. Both of these groups were otherwise similar in terms of alcohol use and exposure to air pollution. The results of the study were unequivocal: smokers of 35 or more cigarettes per day had 40 times the risk of dying from lung cancer than non-smokers (Doll and Hill, 1964). Eventually, smoking prevalence fell to about 5% among the UK doctors (Doll et al., 2005). Presumably, the doctors realized that smoking was not only killing their patients but also them.

In the US, Hammond and Horn (1954) published the results of a cohort study of 180,000 men that concluded that smoking was ‘beyond a reasonable doubt’ a cause of lung cancer. By 1959, Hammond and Horn also established a much larger study of 1 million US adult men and women. The results of this study showed markedly increased risk for men, but importantly, not for women, as the majority of the women had not smoked since early adolescence (Hammond, 1966). The lack of finding an association of smoking with lung cancer in women was used by the tobacco industry to argue against a causative role of smoking for disease (U.S. Department of Health, Education, and Welfare, 1964).

Two landmark government reports summarized the cumulative evidence. The 1962 Royal College of Physicians in the UK documented strong association between smoking and lung cancer, other lung diseases, heart disease and gastrointestinal problems (Royal College of Physicians, 1962). The 1964 US Surgeon General, Luther Terry, released the Surgeon General's Advisory Committee on Smoking and Health (U.S. Department of Health, Education, and Welfare, 1964). This was one of the first ‘exhaustive’ reports, covering more than 7,000 articles relating to smoking and disease in the biomedical literature. It concluded that cigarette smoking was a cause of lung cancer and laryngeal cancer and chronic bronchitis in men and a probable cause of lung cancer in women.

Subsequent to these two reports, there have been periodic systematic assemblies of global evidence on the hazards of smoking as noted above.

### Importance of prolonged smoking to disease risks

The mid-century evidence on the disease risks attributable to smoking was not taken seriously, even in the countries where it was generated. This was in part because of the potentially misleading delay of several decades between cause and full effect. Increased mortality from smoking requires early uptake and continued smoking. Hence, there was a delay of up to 50 years from when the young men in any particular country took up smoking (followed by the young women taking up smoking, a decade or two later) and the time when these studies could document the main hazards in middle ages for various diseases (most notably lung and other cancers, and emphysema). However, there is a shorter latency between smoking and vascular diseases (Jha and Peto, 2014).

**Figure 4.**
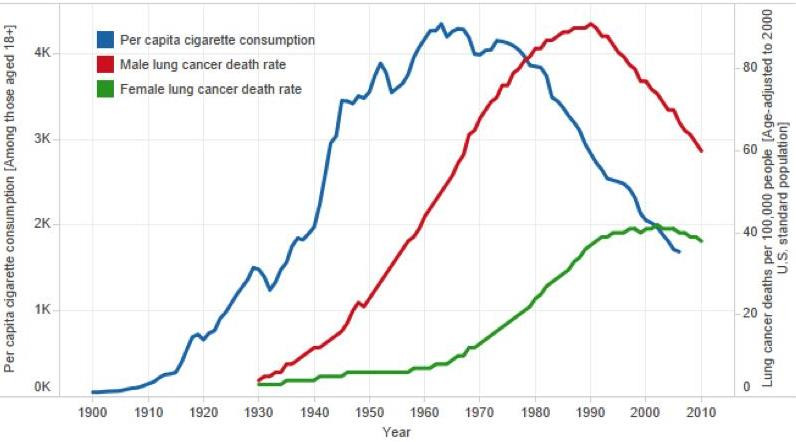
Trends in per capita cigarette consumption and age-standardized lung cancer death rates in the US. Adapted from American Cancer Society (2013).

In the US, cigarette consumption averaged 1, 4 and 10 per-day, in 1910, 1930 and 1950, respectively, after which it stabilized and subsequently fell (Forey et al., 2016; Peto and Lopez, 2001). Peak lung cancer death rates did not however occur until after 1990 in US men and about 2005 in US women (Figure 4). Indeed, measurement of the full effects of prolonged smoking from adolescence to middle-age may require 100 years to observe at the population level (Thun et al., 2013). For example, the full effects of prolonged male smoking (without cessation) were reliably documented only in 2005, among UK doctors born between 1900 and 1930, who were tracked and followed for mortality until the last re-survey in 2001. The UK doctors born between 1900 and 1930 represented those with the highest prevalence of smoking as adults and those who smoked from early adult life. In other words, these doctors represented ‘peak exposure’ measured at the population level (Doll et al., 2005).

Smokers who start smoking may not feel any of the major ill effects until years or decades later, making the link of smoking to disease counter-intuitive. This underestimation of smoking-related health risks in adolescence is particularly relevant to the increased risk of developing cancers from smoking in early-adult life. For example, the risk of developing lung cancer is far higher in individuals who begin smoking at age 15 and smoke one pack of cigarettes a day until they turn 45, than those who start at age 30 years and smoke two packs a day until age 45 years (Peto, 1986). In both instances, the total amount smoked is equal, but early and prolonged smoking markedly increases the risk of lung, and likely, other cancers. (Clinicians often determine their patients’ ‘pack years’ of smoking, but this fails to distinguish those at markedly higher risk because they started early).

The two major sources of evidence on prolonged exposure are national lung cancer mortality data or prospective studies that follow smokers and non-smokers for the development of disease. Lung cancer trends are useful in high-income countries, including Canada, which have had high completeness of death certification and reasonably reliable certification of the causes of death by physicians. Death certification and causes of death are more reliable in middle-age (ages 30–69 years) as compared to older ages (age 70 or older). This various reasons for the difference in reliability have been reviewed earlier (Doll and Peto, 1981; Jha, 2014).

The age-specific patterns of lung cancer deaths provide a very useful way to examine the relevance of age-specific smoking. Lung cancer is nearly entirely caused by smoking in high-income countries (exposure to indoor air pollution from solid fuel use does account for a substantial proportion of lung cancer, particularly in women, in China [Liu et al., 1998]), though such exposure has been uncommon in high-income countries for most of the last century). Moreover, careful reviews of lung cancer rates in mostly non-smoking populations (such as women in Asia), and in prospective studies has shown that rates of lung cancer among non-smokers are substantially lower than among smokers and have changed little over the last few decades (Thun et al., 2008).

**Figure 5.**
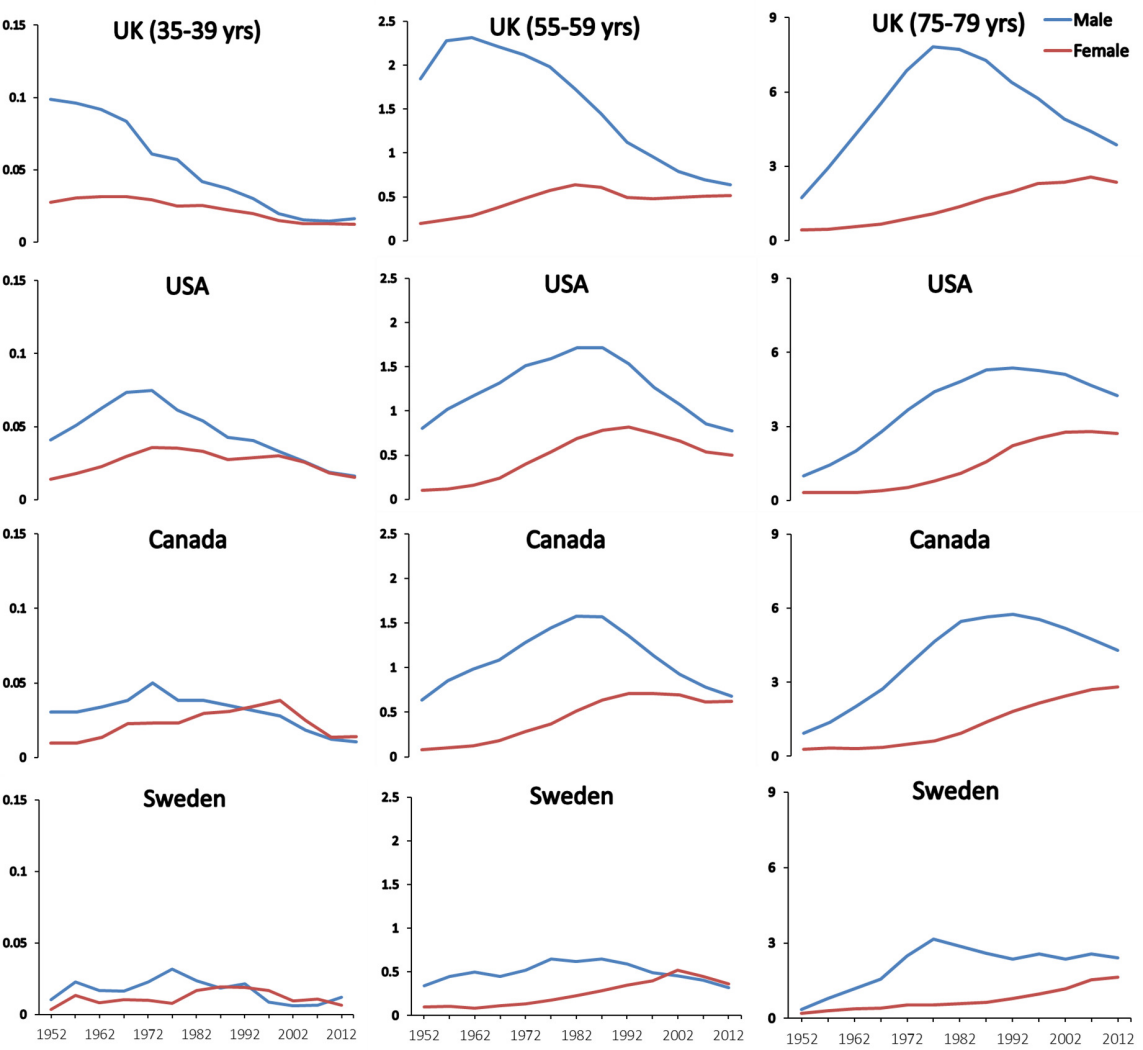
Trends in age-specific lung cancer death rates among men and women at ages 35–39, 55–59 and 75–79 in selected countries.

Closer examination of national lung cancer mortality trends at specific and reasonably narrow ages provides insight on the importance of prolonged and early smoking to subsequent mortality risks. Lung cancer trends can then be related to age-specific different levels and patterns of smoking recorded for different generations. Consider the trends in lung cancer in three age groups separated by two decades (representing roughly one generation): 35 to 39, 55 to 59 and 75 to 79 years. Figure 5 shows that among 35 to 39 year-old and 55 to 59 year-old men, the peak lung cancer death rates occurred around 1970 and 1990 in the US and in Canada, respectively. These men, who died in 1970 and 1990, were born, on average, in 1933 (= 1970–37 and = 1990–57). This means that the typical age of uptake of smoking for these men would have been in the decade after WWII. This represents the period during which a substantial increase occurred in per capita cigarette consumption in both countries (Figure 2). For men aged 75–79, the peak lung cancer deaths in the US and Canada occurred in 1990 and thus correspond to a mean birth year of around 1913. This generation of men in both countries had particularly heavy exposure to high-tar cigarettes and likely smoked in different subtle fashion, in terms of puffing, inhalation, and other features than subsequent generations.

Figures provided by Hangchao Pan and Richard Peto, University of Oxford, 2018.

A more consistent pattern emerges when examining death rates and smoking patterns in the UK. Here, the lung cancer death rates had peaked about two decades earlier among UK men aged 35 to 39, 55 to 59 and 75 to 79 years, representing a mean year of birth of 1903. This also corresponds to the observation that the peak increase in male smoking occurred between WWI and WWII in the UK but during and after WWII in the US and Canada. These data also support the stronger likelihood that most of the cigarettes smoked and the manner of their smoking were more similar over time in the UK than they were in the US (Doll and Peto, 1981).

As a further comparison, men in Sweden never took up smoking at levels observed in other Western countries. This lack of increase in smoking has been attributed mostly to the widespread use of ‘snus’, which provides rapid nicotine stimulus to the brain with similar rapidity as cigarettes, in Sweden (Ramström et al., 2016).

In Swedish men aged 55 to 59 years, the peak lung cancer death rates in Sweden were only a quarter of that seen in UK men. Moreover, the mean age of birth of the men in the three age comparison groups was approximately 1938; meaning Swedish men who took up smoking during so the 1960s.

Similarly, the lung cancer death rates in women peaked at these age groups about two decades later than that of men (and indeed have plateaued in older women aged 75–79 only recently). These findings also correspond to the period in which women typically born around WWII began to smoke heavily in the 1960s.

The robustness of such analyses is of course affected by differences in death certification and coding of lung cancer (Doll and Peto, 1981). In all three countries, lung cancer is a reasonably distinct diagnosis, particularly before older ages (about 70 or 80 years of age), and nearly 100% of deaths from about 1950 onward were medically certified. The trends in the death rates from lung cancer in various age groups from 1960 to 2010 have been reasonably similar in the US and Canada, which is expected given the similarities in the causes of death (World Health Organization, 2016), smoking patterns (Figure 1), and the procedures for certifying both death and lung cancer in the two countries. Note, however that the US peak rates at ages 35 to 39 years in men, are about double that of Canadian men, though similar at ages 55 to 59 years and 75 to 79 years. This may be due in part to smaller numbers of lung cancer deaths in the smaller Canadian population, or, may reflect subtle differences in the type of smoking or undocumented differences in the types of common cigarettes smoked between the two countries (Fischer et al., 1990a; Fischer et al., 1990b). The cohorts of men and women born after 1950 have mostly smoked lower-tar cigarettes than the men who began to smoke either between the two World Wars or just after WWII.

Earlier careful review of US lung cancer death rates in prospective studies of US veterans finds that lung cancer risks are particularly elevated among those who began to smoke significant amounts from early adulthood (Doll and Peto, 1981). The age-specific relationship of smoking to lung cancer is likely similar for selected other cancers, particularly upper aerodigestive cancers, though this relationship likely differs for other diseases made more common by smoking. Prolonged smoking and early initiation appear to be a particularly relevant risk factor for emphysema and chronic lung diseases. Vascular disease is more responsive to short-term effects given the role of smoking in causing vascular spasm and in the shorter time period for development of atherosclerotic plaques, which cause heart attacks and strokes.

**Figure 6.**
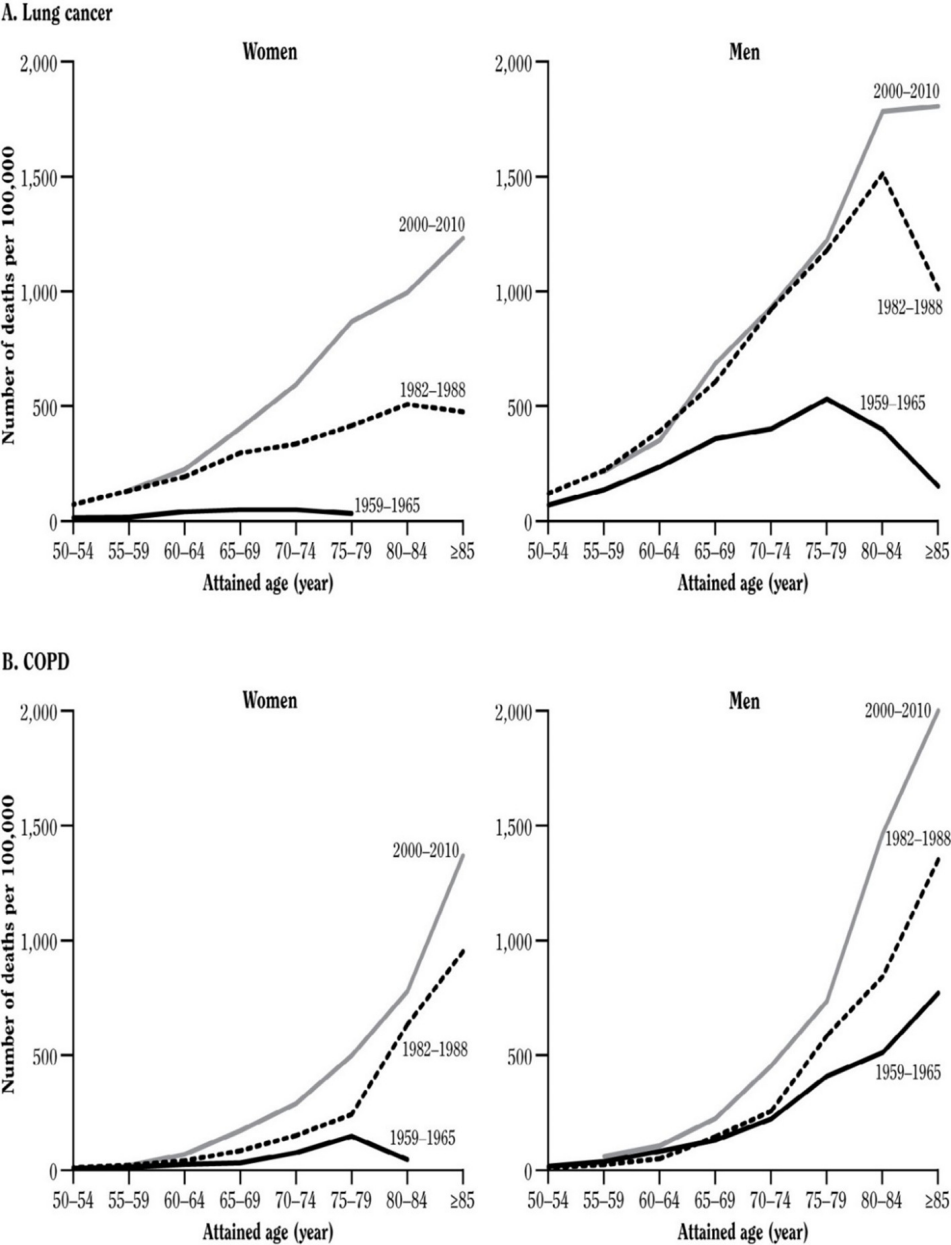
Lung cancer death rates by sex by study period. Adapted from U.S. Department of Health and Human Services (2014).

The peak mortality effect of smoking among men occurred in most high-income countries in the last quarter of the 20^th^ century. The full effects of persistent smoking on premature mortality in women can be assessed only in the first quarter of the 21^st^ century. In the US, the lung cancer death rate among women who never smoked has been low and approximately constant for many decades, while the lung cancer rate among women who smoke has been increasing steeply. The US female lung cancer death rate ratio (current-smoker versus never-smoker), has increased greatly over the last half-century (Figure 6). In the 1960s, it was 3-fold; in the 1980 s, 13-fold and in the 2000 s, 26-fold (similar to the death rate ratio among men in the US [Thun et al., 2013] or among men or women in the UK [Pirie et al., 2013]).

This is because US women aged about 60 years who were smokers in the 2000s had smoked since early adult life, whereas women who were smokers in the 1960s had not. Similar relationships are seen for chronic lung-disease. The key implication is, of course, that the hazards among men and women from various diseases are now comparable among women and men who start early in life and do not quit smoking.

### Quantifying mortality from smoking in the US, UK and Canada from 1960 to 2020

Peto et al. (1992) developed a method that provides indirect estimates of tobacco-attributable mortality across countries which have reasonably high coverage and quality of routine death certification, including most high-income countries. This is based on the observation that most lung cancer deaths occur among smokers, and that non-smoker lung cancer rates are comparably low across most high-income countries and have been mostly unchanged over the last few decades (Thun et al., 2008). Lung cancer is then used as an indicator not only of the extent to which smoking causes lung cancer, but also to what extent if that particular population is affected by smoking exposure. This involves indexing the absolute lung cancer deaths (subtracting the low rates among non-smokers) to the relative risks from the large US prospective studies (effectively very similar to the USSGR relative risks shown in Appendix 1—table 1). This allows a reasonable estimate of the extent to which cancers other than lung, respiratory disease, ischemic heart disease, stroke and other causes of death are caused by smoking.

Use of lung cancer as an index of smoking is a crude method but is reliable over time, and useful in settings that meet these conditions such as by social strata (Jha et al., 2006) and including men in north Mexico, but not in South Mexico (Reynales-Shigematsu et al., 2018). The Peto estimates (updated to 2015; Peto et al., 2018) provide totals for 1955 to 2015 and I interpolated the annual results using the trends per decade. I did backward calculations to 1950 using the same annual rate of change as documented between 1955 and 1965. 

These reveal that cummulatively from 1960 to 2020, there were about 29.5 million, 9.3 million and 2.6 million deaths from smoking in the US, the UK and Canada, respectively (Table 2) or a total of 41.3 million adult deaths. Over 60% of these deaths occured in males, and these collectively represented about 22% of all adult deaths in these three countries. Of these deaths, about 40% were between ages 35-69 years, comprising about 16 million deaths, with an average loss of life of about 20-25 years. The remainder of the deaths occured after the age of 70 years.

**Table 2.** Trends in smoking-attributable deaths from 1955 to 2015, (with projections to 2020) by sex in US, UK, and Canada (in thousands).

Year	US		UK		Canada		
	Smoking-attributable	Total	Smoking-attributable	Total	Smoking-attributable	Total	
**Males**							
1955	139	882	90	303	8.8	75	
1965	235	1 036	126	324	17	87	
1975	305	1 063	142	337	26	96	
1985	335	1 092	125	325	31	100	
1995	341	1 163	95	308	33	111	
2005	302	1 199	67	279	29	116	
2015	286	1 325	55	264	28	129	
1960–2020*	18 040	68 760	6 100	18 370	1 640	6 390	
**Females**							
1955	1.4	663	12	285	0	54	
1965	19	795	24	307	0.7	61	
1975	79	858	43	331	4.2	70	
1985	169	983	59	331	11	80	
1995	272	1 135	69	332	20	99	
2005	302	1 229	66	306	26	113	
2013	302	1 288	60	277	30	126	
1960–2020*	11 430	62 880	3 210	18 840	919	5 490	
Both sexes 1960–2020*	29 470	131 640	9 310	37 210	2 559	11 880	

^* 1960-2020^* 1960–2020 totals by taking totals for 1965, 1975, 1986, 1995, 2005 and 2015 totals multiplied by 10. *Note.* Author’s calculations based on Peto et al. (2018). Cumulatively, from 1960 to 2020, smoking killed about 29.5 million Americans, 9.3 million UK residents, and 2.6 million Canadians, or a total of 41.3 million adults (Table 2).

Globally, there were about 6 trillion cigarettes consumed worldwide, of which about a third are consumed in China alone. Global consumption has increased from about 5 trillion cigarettes in 1990 (Jha and Peto, 2014). For the US, UK and Canada I obtained cigarette sales data from global smoking sales statistics (Forey et al., 2016). Under tobacco reporting regulations by the federal government of Canada, tobacco manufacturers and importers must give Health Canada annual reports that include sales data manufacturing information and product information. These data are publicly available on an aggregated industry basis. As such, the reporting of sales of tobacco in Canada over time is more reliable versus that of other countries. I lagged the ratio of deaths to cigarette smoke by 20 years to take into account the delay between the uptake of smoking and the development of disease as I have discussed above.

**Figure 7.**
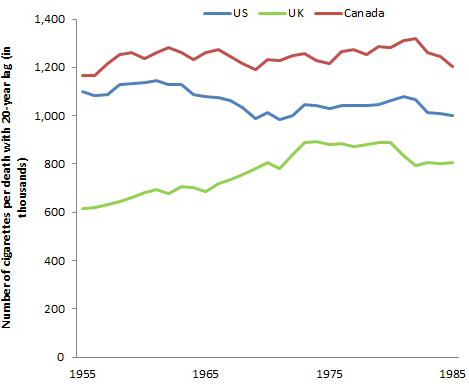
Number of cigarettes (from 1955 to 1985) per death with a 20 year lag in US, UK, and Canada (from 1975 to 2005). *Note.* Author’s calculations.

During this same period, cigarettes sold in the US, UK and Canada were about 32.6 trillion, 7.0 trillion and 3.2 trillion, respectively. Using the 20 year lag between smoking and disease development as a reference point, the relationship of total cigarette sales in Canada and in the US suggests approximately every 1.0 to 1.2 million cigarettes smoked yielded one death (Figure 7). This estimate is consistent with another published in 2014 using global sales (Jha and Peto, 2014). However, for the UK, the relationship shows that every million cigarettes smoked yielded 1.3 deaths. This might reflect the composition of cigarettes, and the use of higher-tar cigarettes in the UK with the peak of smoking occurring prior to WWII.

In 2014, cigarettes sales in billions in the US, UK and Canada were 263, 51 and 30, respectively, each of which in absolute terms is a substantial decline from the peak annual sales. Nonetheless, the absolute sales have remained steady since about 2005 in Canada, but with continuing declines in the US and UK. An increase in sales in Canada from 1993 to 1995 was in response to a well-funded tobacco industry effort to smuggle its own products and to force a reduction in the tax rate (World Bank, 2019). The effects of the smuggled cigarettes, both in direction consumption but also in reducing prices of legal cigarette (which raised consumption) were approximately 30 to 40 billion excess cigarettes over a decade. Hence, eventually, about 30,000-40,000 Canadians will be killed from this excess consumption (Jha et al., 2020). 

These sales do not, of course, adjust for population size and growth. However, that is deliberate. If the goal is to quantify the extent to which cigarette sales translate into future deaths, then the absolute sales totals and absolute death totals is a relevant statistic.

## Smoking risks for total mortality and for specific conditions

### Key messages for the individual smokers

The main messages for smokers, based on the contemporary epidemiological evidence are three-fold (Box 1).

Box 1. Three main implications for individuals who become cigarette smokers in adolescence or early adult life.

1. The risk for disease development is big, if they continue smoking.
  - Continued smoking eventually kills at least half of men and women who smoke. Among persistent cigarette smokers, whether men or women, the overall relative risk of death throughout middle-age and well into old age is at least twofold higher than otherwise similar never-smokers. Among smokers of a given age, more than half of those who die in the near-future would not have done so at never-smoker death rates.
  - On average, smokers lose at least one decade of life. This average combines a zero loss for those not killed by tobacco with the loss of much more than one decade for those who are killed by it.
2. At least half of those killed are middle-aged (ages 30–69 years), losing many years of life.
  - Some of those killed in middle-age might have died anyway, but others might have lived on for another 10, 20, 30, or more years.
  - On average, those killed in middle age lose about 20–25 years of never-smoker life expectancy.
3. Smoking cessation works to reduce health risks.
  - Those who stop smoking before age 40 avoid more than 90% of the excess risk among those who continue to smoke. Those who stop smoking before age 30 avoid nearly all of the smokers’ excess risk.
  - Those who have smoked cigarettes since early adult life but stop at 30, 40, 50, or 60 years of age, gain, respectively, about 10, 9, 6, and 4 years of life expectancy, compared with those who continue smoking.

Source: Author’s calculations from various citations.

Here, I elaborate on the first two of these messages. A following section on cessation provides greater details of the benefits of cessation.

**Figure 8.**
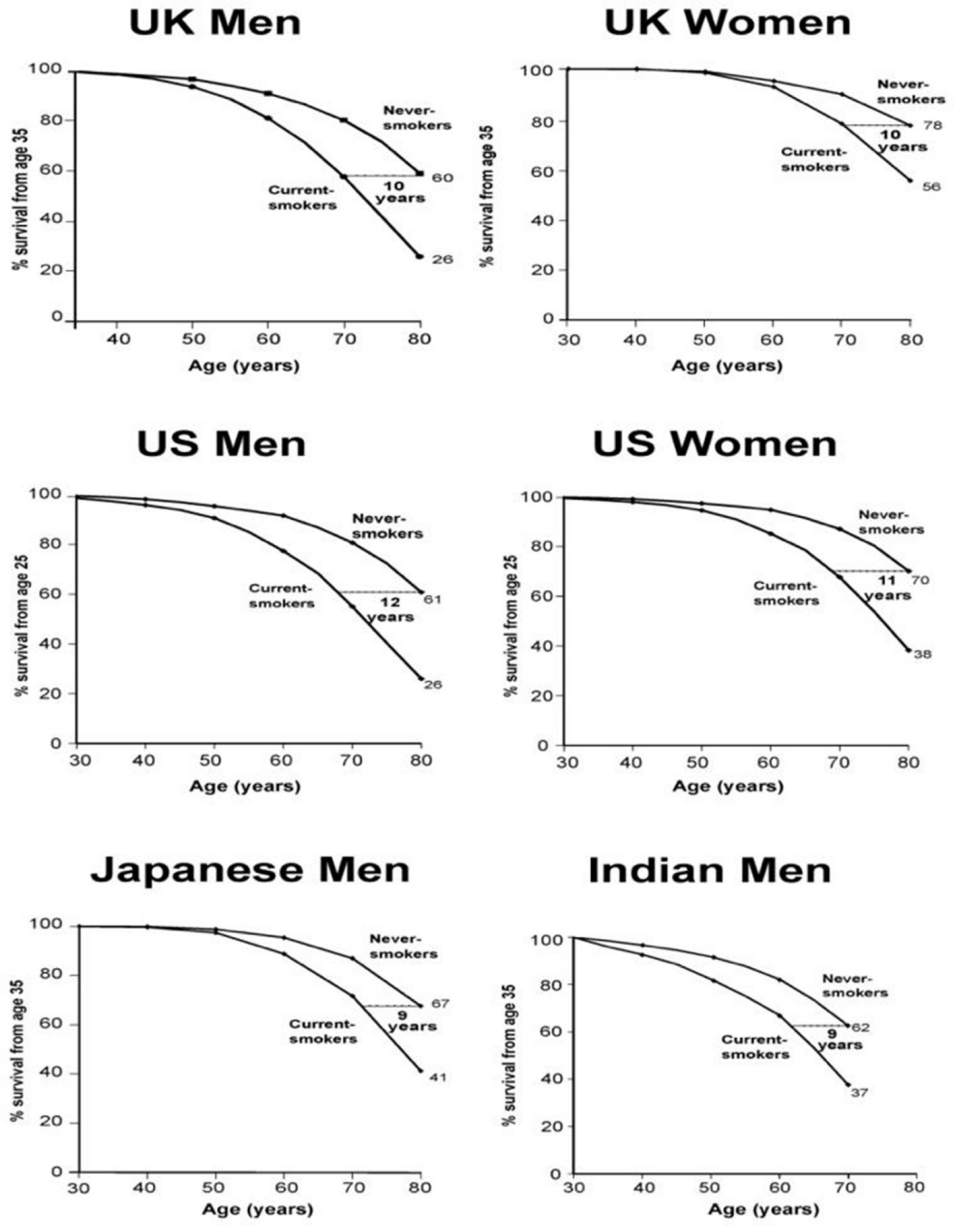
Survival differences between smokers and non-smokers, adjusting for various factors. Adapted from Jha and Peto (2014), p. 62.

First, the risk is big. Large epidemiological studies in the UK (Doll et al., 2005; Pirie et al., 2013), US (Jha et al., 2013; Thun et al., 2013), Japan (Sakata et al., 2012), and India (Jha et al., 2008; Jha et al., 2015) have examined the eventual effects on mortality in populations where many began to smoke cigarettes seriously in early adult life and did not quit smoking (Figure 8). Among persistent men or women cigarette smokers, the overall relative risk of death throughout middle age and well into old age is about two to three-fold higher than otherwise similar people who never begin smoking. Among smokers of a given age, more than half of those who die in the near future would not have done so at the rate of people who never smoked.

This increased risk is now seen among women who began smoking early in adult life and did not quit, chiefly those women born around WWII. The loss of a full decade of life is seen, surprisingly, among male cigarette smokers in India, despite the later age of uptake and generally fewer cigarettes smoked (Jha et al., 2008). These findings further suggest that 3-fold higher death rates among persisting smokers represents that of a fully-mature smoking epidemic, given the fact that the late 20^th^ century risk among women also reached 3-fold of that versus non-smokers, as observed for men two decades earlier.

Secondly, many of those killed are of middle age. On average, those killed at ages 35–69 years lose 23 years of life in the US, UK or Canada (Peto et al., 2018). This continued difference in risk throughout middle age and into old age leads to an overall reduction in survival by an average of one decade (Figure 8). The decade of lost life expectancy for typical smokers combines a zero-loss for some of the smokers who are not killed by smoking and about a 20 to 25 year loss for those smokers who are killed by smoking.

Although there has been a decrease in the amount of smoking in recent years in US, UK and Canada as noted in Table 1 the contemporary epidemiological evidence finds that smoking as little as five cigarettes a day is substantially hazardous (Pirie et al., 2013). Despite the reduction in smoking amount, smoking will continue to be a major cause of excess mortality among the significant minority of adults that remain smokers.

Similarly, careful reviews (Doll and Peto, 1981), and recent use of indirect-based methods that rely on lung cancer mortality to estimate smoking-attributable deaths (Peto et al., 1994), find that the age-standardized rates of cancers not attributable to smoking are falling in Canada (and in nearly all high-income countries). This is contrary to popular misperceptions about an ‘epidemic’ of cancer.

The 21^st^ century evidence suggests that there is an eventual risk of about three-fold mortality rate versus that of non-smokers, corresponding to about two-thirds of smokers being killed eventually by their addiction. Hence, the effect on total mortality is an appropriate starting point to quantify smoking hazards in high-income countries, which is supported by the specific evidence on particular diseases.

### Key diseases attributable to smoking

The evidence for the range of diseases caused by smoking has expanded considerably since the early studies focusing mostly on lung cancer. Importantly, the leading causes of death that are due to smoking are also the major diseases that, even in non-smokers, constitute the leading causes of death in US, UK and Canada. In each country, vascular, neoplastic, and respiratory disease collectively accounted for about 75% of all current adult deaths from all causes (World Health Organization, 2016).

Given the strong evidence that at every age, smokers have about a two to three-fold higher death rates versus otherwise similar non-smokers, the key issue here is to analyse if the specific conditions that contribute to higher overall excess risk of death is, in fact, due to smoking. This in turn requires scrutiny of all the scientific evidence linking smoking with particular disease and careful analysis of ‘negative’ studies that do not find an association between smoking and disease or, indeed, find that smokers have lower rates of disease than non-smokers. To avoid ‘publication bias,’ where positive results linking smoking and disease are published, but negative studies that do not support this link remain unpublished, researchers have outlined methods of comprehensive searches of all available scientific literature, and statistical methods to ascertain the likely extent of negative studies sitting on the shelf, impacting final conclusions (IARC Working Group on the Evaluation of Carcinogenic Risk to Humans, 2004; U.S. Department of Health and Human Services, 1989). Of course, the publication bias would almost certainly go in the other direction for most internal tobacco-industry studies, many of which remain outside searchable scientific arenas.

**Figure 9.**
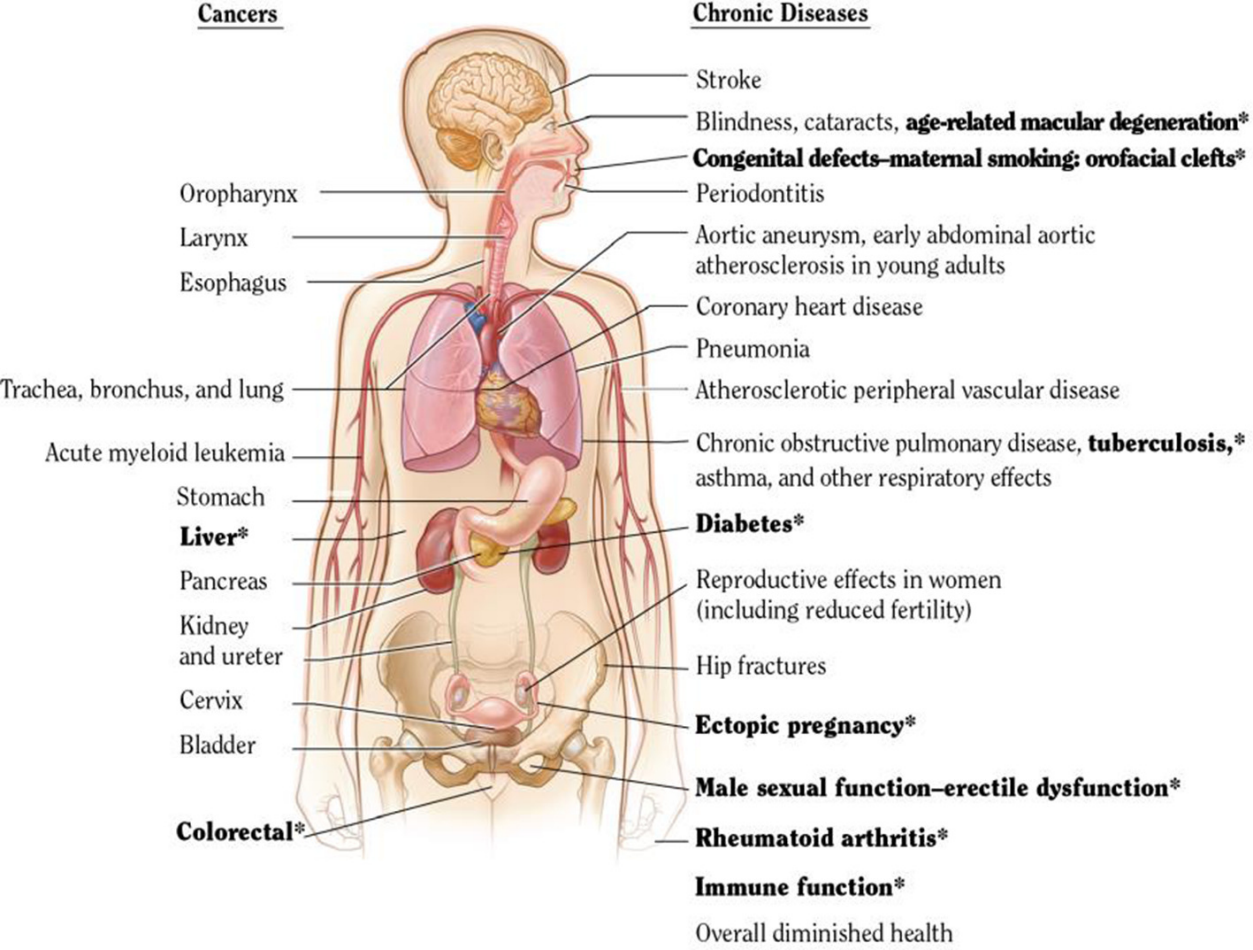
USSGR 2014 estimation of anatomic sites and cancers linked to smoking. Adapted from U.S. Department of Health and Human Services (2014).

The US Surgeon General and the IARC have periodically assembled expert groups for such systematic reviews. These expert groups use slightly different criteria to assess the strength of the evidence. The USSGR definitions use three levels: ‘sufficient evidence’ in terms of the risk of a particular disease to smoking (or, less commonly, a protective effect of smoking), suggestive evidence, or insufficient evidence. Figure 9 shows the adult anatomic sites and cancer sites of which the 2014 USSGR linked to smoking. The bolded text represents those conditions, which are newly deemed to have ‘sufficient’ links to smoking.

The list of tobacco-attributable conditions is expanding over time, as highlighted in the 2014 USSGR. This suggests that overall mortality is a robust and valid metric to estimate tobacco-attributable risk (particularly, that since there are very few diseases reduced by smoking, a focus on total mortality is not misleading). Note further that for most conditions there is a strong correlation between death and disability (Menon et al., 2019), with only a few exceptions, such as loss of teeth or rheumatoid arthritis, conditions that cause far more disability than mortality. The use of mortality totals is likely to also reflect also on disability totals.

### Avoidable proportion of major diseases at non-smoking death rates

The 2014 USSGR published for relevant age groups and for men and women, the relative risks (RR) for various conditions, after adjusting for differences in age, alcohol use, obesity, education or some related measure of social status. This full table is attached as Appendix 1—table 1.

The main sources of the USSGR estimates include the second US prospective cancer study, which surveyed one million Americans, the CPS II study, and the results from five pooled studies of more contemporary cohorts, represent the most recent period (2000–2010). These include the National Institutes of Health–American Association of Retired Persons Diet and Health Study (Schatzkin et al., 2001), the American Cancer Society CPS II Nutrition Cohort (a subset of the original CPS II mortality study [Calle et al., 2002]), the Women's Health Initiative (Paskett et al., 2007), the Nurses' Health Study (Holmes et al., 2007) and the Health Professionals Follow-up Study (Kenfield et al., 2011). These represent US results, but similar results have been reported in Canada with smaller sample sizes (Manuel et al., 2012). Similarly, among women and men the nationally representative US National Health Interview Study reported similar relative risks for lung cancer, vascular and respiratory disease (Jha et al., 2013), as did the Million Women Study in the UK among women (Pirie et al., 2013). Finally, the prospective study among Japanese Atomic survivors also reported similar relative risks (Sakata et al., 2012). For the most part, the smoker: non-smoker relative risks were unaffected by adjustments for other risk factors in most of the studies, even though smokers tend to drink alcohol more commonly, and have lower education levels.

Table 3 provides the avoidable proportion of deaths for each major disease. This is calculated by (RR-1)/RR with the RRs derived from the USSGR report of 2014. This analysis shows that among smokers, over 90% of deaths from lung cancer at various ages or chronic obstructive deaths at ages 65 or older would have been avoided at non-smoking death rates, reflecting the very high relative risks of smoking for these conditions. About half to four-fifths of coronary heart disease deaths among smokers would have been avoided; the proportion avoidable was even larger in younger adults. Approximately a third to half of stroke deaths among smokers would have been avoided. Overall, up to two-thirds of all deaths among smokers would have been avoided at non-smoking death rates.

**Table 3.** Proportion of deaths by cause among current smokers that would be avoided at non-smoking death rates, by sex and age.

	Among smokers, % avoided at non-smoking death rates								
Disease/sex	Males				Females				
Age groups	35–54	55–64	65–74	≥75		35–54	55–64	65–74	≥75
Lung cancer	93%	95%	96%	96%		92%	95%	96%	96%
Other cancers	43%	46%	57%	54%		22%	52%	51%	48%
Coronary heart disease	74%	67%	64%	49%		80%	69%	70%	56%
Cerebrovascular disease			54%	32%				56%	41%
Aortic aneurysm, other arterial and atherosclerosis			86%	80%				85%	83%
Diabetes mellitus			33%	9%				35%	9%
All vascular at ages 35–64	58%	60%				59%	49%		
Influenza, pneumonia, tuberculosis			61%	38%				43%	51%
Chronic obstructive pulmonary disease			97%	96%				97%	95%
All respiratory at ages 35–64	78%	93%				84%	89%		
All causes	61%	66%	67%	58%		44%	62%	65%	60%

Notes: Author calculations. The avoidable proportion for each condition and sex is calculated as (RR_c_-1)/RR_c_, where RR_c_ refers to the smoker: non-smoker relative risks (RR) for current smoking in the U.S. Department of Health and Human Services (2014) (Appendix 1).

The avoidable proportion should be taken as conservative, as the relative risks in the USSGR report may be underestimates. The prospective studies had enrolled smokers and non-smokers before they developed disease, and smoking status was collected only at this baseline. Some of those who reporting smoking at baseline would have quit subsequently, as there have been, in recent years, increases in cessation by older adults. This cessation would reduce their risk of death, bringing them closer to the observed mortality rates of non-smokers. Had they not quit, the observed differences in relative risks between smokers and non-smokers would most likely have been larger.

### Understanding the contemporary risks: specific conditions

#### Cancers, vascular and respiratory disease

Cancers: The USSGR (U.S. Department of Health and Human Services, 2014). and IARC Reports (IARC Working Group on the Evaluation of Carcinogenic Risk to Humans, 2004) have previously concluded that there are sufficiently strong associations to define tobacco use as a cause of several cancers including lung, tongue, lip, larynx, oropharynx, bladder, kidney, oesophagus, stomach, pancreas, cervix and liver.

The 2014 USSGR also found that existing strong evidence for smoking as a cause of squamous cell lung cancer in men is strongly enforced by recent evidence of these links in women, who typically begin smoking later in life. Moreover, among smokers, the risk of developing adenocarcinoma of the lung has risen since the 1960s. This increased risk is likely the result of changes in the design and composition of cigarettes, which might include use of ventilated filters (leading to deeper inhalation of tobacco smoke), and perhaps changes in nitrosamines levels since the 1950s. There is sufficient evidence for a causal relationship between smoking and hepatocellular cancer. The report noted a suggestive relationship of smoking to adenomatous polyps and colorectal cancer. Smoking was found not to be a cause of new prostate cancers, though it is important to note that smokers do have high risks of death if diagnosed with prostate cancer, as well as a greater chance of more advanced and poorly differentiated cancers.

Cardiovascular disease, including coronary artery disease, stroke, aortic aneurysm, and peripheral artery disease, is the leading cause of death in most countries worldwide. Hence, while the relative risks of smoking for specific vascular diseases are smaller than that for cancers and respiratory disease, vascular mortality dominates the absolute burdens of tobacco-attributable diseases in many countries.

The chief finding from the 2014 USSGR Report and the more contemporary cohort studies is that the smoker: non-smoker mortality risks for ischemic heart disease have become more extreme over time. This is due in part to rapid improvements in the treatment of vascular disease that have contributed to rapidly falling background rates among non-smokers. By contrast, smokers are not receiving the full benefit of technologies that benefit non-smokers (Jha et al., 2013).

The relative risks for non-fatal heart attack are greater than those for fatal heart attack (Table 4). This would suggest, for example, that the true population burden of smoking-attributable ischemic heart disease for the number of hospital admissions is much larger than that derived from mortality studies. Moreover, the relative risks are greater at younger ages, so that at ages 30–39, about 80% of the heart attacks in UK men can be attributed to smoking (Parish et al., 1995). Naturally, the absolute rates are greater at older ages.

**Table 4.** Relative risks for fatal and non-fatal ischemic heart disease by age for men.

STUDY	AGE (years)	Relative risk
*Fatal ischemic heart disease*		
US	35–69	2.5
CHINA	35–69	1.2
INDIA	30–69	1.6
*Non-fatal ischemic heart disease*		
UK	30–39 40–49 50–59 60–69 70–79	6.3 4.7 3.1 2.5 1.9
INDIA	Current Ever Former	4.7 3.9 2.6
SEVERAL COUNTRIES	Current Former	3.0 1.9

*Note.* Author’s calculations based on an earlier review (Jha et al., 2010).

For respiratory disease, including emphysema and chronic bronchitis, there have also been substantial increases in mortality among smokers in the last two to three decades, with the smoker: non-smoker hazards becoming particularly extreme among women (Thun et al., 2013). The 2014 USSGR also identified that smoking as a cause of tuberculosis death and recurrent tuberculosis, but stated that evidence was not sufficient to evaluate if smoking causes infection. While tuberculosis deaths and infection are uncommon in Canada, they remain a major cause of death in low and middle-income countries (Bates et al., 2007; Gajalakshmi et al., 2003). Smoking compromises the immune system, which leads to increased risk of pulmonary infection, as well as loss of voice.

#### Other outcomes

The 2014 USSGR added newer conditions that were not listed as being causally related to smoking in the 2004 USSGR. Maternal smoking in early pregnancy is a cause of orofacial clefts and might be linked to clubfoot, gastric and vascular malformations. Maternal smoking might be linked to behavioural disorders and attention deficit in children. Smoking is a cause of ectopic pregnancy, and of erectile dysfunction in men. Rheumatoid arthritis, congenital effects and colorectal cancer are possibly attributable to smoking.

#### Diseases not attributable to smoking or protected by smoking

Some conditions are not sufficiently proven to be caused by smoking, or are (less commony) reduced by smoking. These include Alzheimer’s disease, breast cancer, inflammatory bowel disease and uterine cancer. A popular myth is that smoking protects against dementia and Alzheimer’s disease. However, the UK doctor’s prospective study (Doll et al., 2005) noted that dementia risks were unaffected by smoking history. A systematic review of about 50 epidemiological studies found that smoking modestly raised the risk for Alzheimer’s but had no effect on the development of dementia (Peters et al., 2008). The 2014 USSGR noted that there is some evidence that smoking reduces the risk of endometrial cancer in women. Among the inflammatory bowel diseases cases, smoking might reduce ulcerative colitis but raise the risks of Crohn’s disease. Finally, the 2014 USSGR found that there was no definitive evidence that smoking causes breast cancer.

The presence of some diseases not attributable to smoking strengthens the argument about the causal nature of the link between smoking and specific diseases. The strength of association of various conditions to smoking also varies, as defined by the relative risk of death between otherwise similar smokers and non-smokers. For example, there is a strong and consistent relationship of tobacco smoking to lung and many upper digestive cancers. By contrast, the relationship between smoking and colorectal cancer is weaker. However, the excess risk for almost all of these conditions is likely causal. Thus, the strength of the association is less relevant for public health action than is the list of the most common conditions in the population that accrue the largest absolute number of deaths.

## Reductions in total and in cause-specific mortality from smoking cessation

In contrast to the long delay between smoking onset and the development of disease, the main effects of widespread cessation are seen much more rapidly. Worldwide, cessation is the only practicable way to avoid a substantial proportion of tobacco deaths before 2050 (Jha and Chaloupka, 1999), as a substantial reduction in uptake by adolescents will have its main effect on mortality rates after 2050.

### Cessation trends in US, UK and Canada

**Figure 10.**
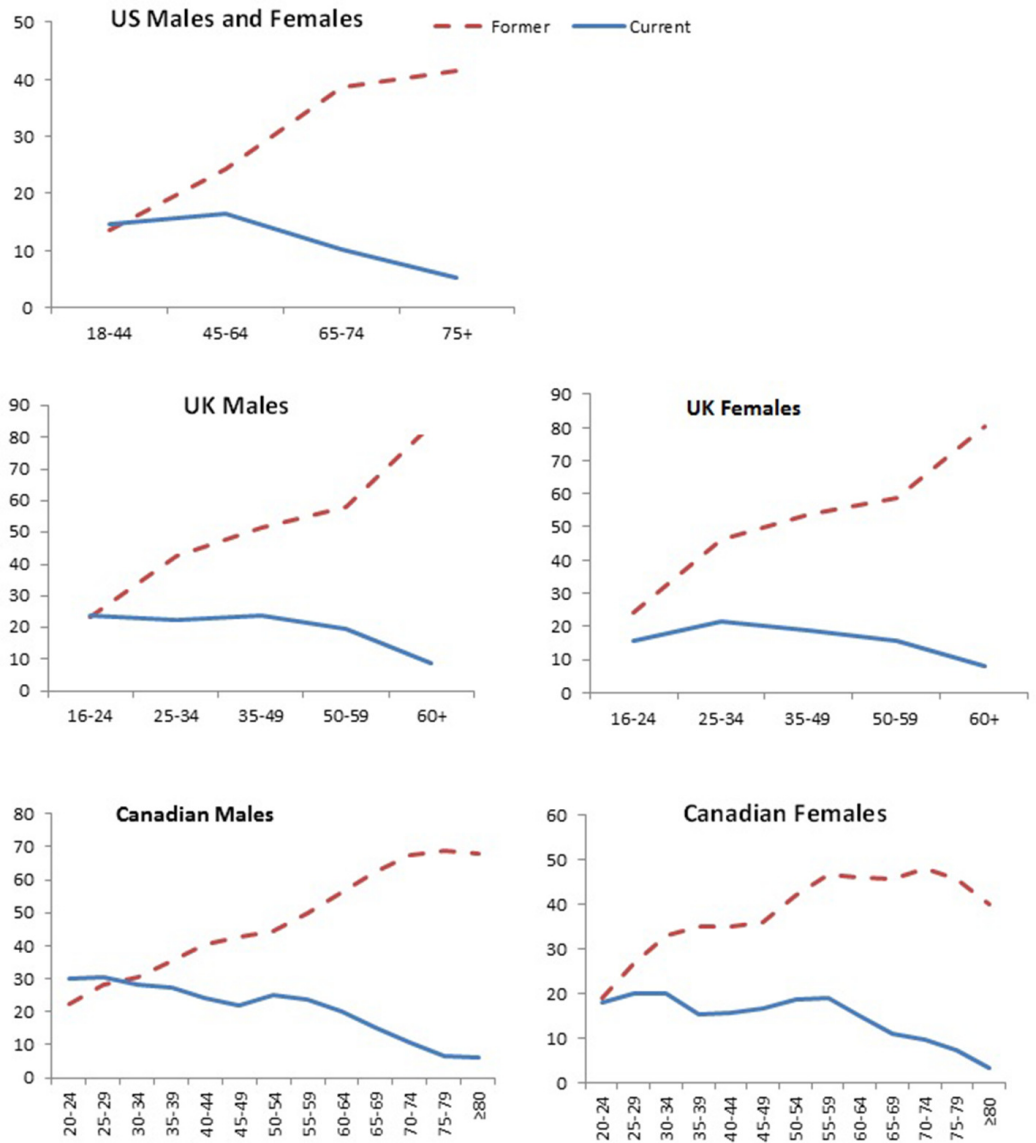
Former and current smoking prevalence, US (2017), UK (2017), and Canada (2014) by sex and age group. *Note.* Data from National Center for Health Statistics, 2017, *NHIS*, Office for National Statistics, 2017, *Adult Smoking habits in Great Britain*; Statistics Canada, 2016, *Canadian Community Health Survey.*

The prevalence of former smoking in middle-age is a useful measure of the success of tobacco control. Currently in Canada, the US and the UK, there are as many former as current smokers between the ages of 45 to 64 years (Figure 10) The short-term relapse rates among smokers considering cessation is very high. However, among those who persist in cessation, few re-start smoking.

There is a large gap in high-income countries between the intent to quit smoking and actual success rates. This is mostly a consequence of the strongly addictive nature of cigarettes. Two-thirds of smokers wish to quit, while only about half of that actually try, while far fewer succeed (U.S. Department of Health and Human Services, 2020). In 2015, nearly two-thirds (65.8%) of Canadian smokers were seriously considering quitting in the next six months. Of those, about half (48.2%) were considering quitting within the next thirty days, which was equivalent to 31.1% of all current smokers. Between 1999 and 2015, the percentage of Canadian smokers seriously considering quitting in the next six months appears to have increased slightly (Reid et al., 2017). In 2015, over half (52.3%) of Canadian smokers and recent quitters reported having made at least one attempt at successful cessation in the past year, while more than one-third had made multiple attempts. From 1999 to 2015, the percentage of smokers and recent quitters who had attempted to quit in the past 12 months appears to have remained fairly stable, at around half (Reid et al., 2017). The US and the UK reports similar patterns of attempted cessation (Babb, 2017; Office for National Statistics, 2018b).

### Reduction in overall mortality from cessation

Smokers who stop smoking before age 40 (preferably well before age 40), avoid more than 90% of the excess risk for overall mortality among those who continue to smoke. Those who stop before age 30 avoid 97% of the risk of death. Those who have smoked cigarettes since early adult life but stop at 30, 40, or 50 years of age gain, respectively, about 10, 9, and 6 years of life expectancy, compared to those who continue smoking (Jha et al., 2013; Pirie et al., 2013).

**Figure 11.**
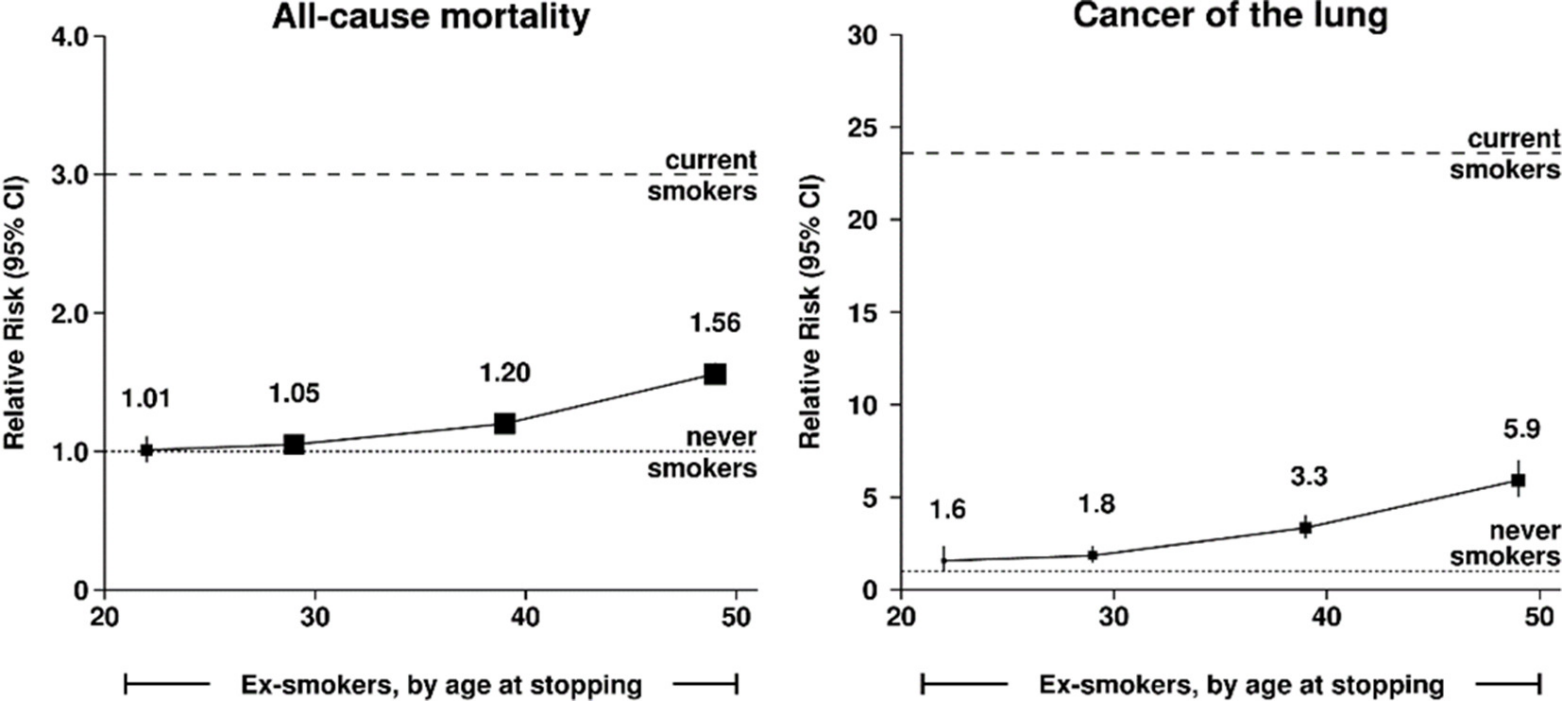
Relative risks for all-cause mortality and cancer of the lung, by age of quitting, among UK women. Adapted from Pirie et al. (2013), p. 138.

The reductions in smoking risk for all deaths and for lung cancer mortality among women in the UK are shown in Figure 11. Very similar results were seen in the US for these same age groups among men and women (Jha et al., 2013). The smoker/non-smoker risks quickly converge for vascular disease, for some cancers other than lung, though less quickly for lung cancers, with intermediate convergence for respiratory disease. The overall persisting hazard for death from any cause is reduced but not eliminated. For those who smoke until age 40 years and then stop, the remaining excess risk of about 20% (relative risk, 1.2, hence 120% risk less 100% baseline risk = 20% excess risk) is substantial and implies that about one in six of these former smokers who dies before the age of 80 years would not have died if their death rates had been the same as otherwise similar non-smokers. However, this 20% excess has to be compared to an excess risk of 200% if they continued to smoke (RR = 3, hence 300% risk less 100% baseline risk = 200% excess risk). Similarly, for lung cancer, quitters by age 40 retain a substantial excess risk of 230%, but this is dwarfed by the 2000% excess risk from continued smoking. In both cases, the relative reduction in excess risk among former versus current smokers exceeds 90%.

Similar results on reductions in lung cancer risk in former smokers are seen in other settings. The excess lung cancer mortality avoided in men who stopped smoking by age 40 was 91% in Germany and 80% in Italy (Crispo et al., 2004). In countries such as Canada, the UK, US or Poland, where high mortality rates from smoking were followed by widespread cessation, mortality from smoking has greatly decreased, for example by 25% per decade in Canada (Peto et al., 2018; Jha, 2009).

Earlier reviews note that the relationships of excess-risk for overall mortality with halving smoking amount reduces mortality risk far less than cessation (U.S. Department of Health and Human Services, 2014). This would imply that the mean reduction in daily amount smoked in the three countries from 1999 to 2013 (about three cigarettes fewer, per day, per smoker; Table 1) has had only a minimal impact on the risk of death. Cessation remains far more important than reduced smoking amount.

### Reduction in specific diseases from cessation

The US Surgeon General has issued a series of reports on the health benefits of smoking cessation starting in 1990 (U.S. Department of Health and Human Services, 1990). Those conclusions were updated in subsequent reports in 2001, 2004, 2006, 2010, 2014 and 2020. A brief summary of the effects of cessation on cardiovascular disease, cancers and respiratory disease follows.

Cardiovascular disease: In patients with existing heart disease who smoke, cessation significantly reduces all-cause mortality, deaths due to cardiac causes, sudden death and the risk of both new and recurrent cardiac events. The reduction in risk of recurrence or death is estimated to be about 30–45%. Smoking cessation benefits people at any age, but the benefits are greater at younger ages compared with older ages (Parish et al., 1995). The risk of coronary heart disease falls rapidly after cessation and then declines more slowly. The excess risk falls by about half after five years of cessation and then gradually approaches the risk of people who have never smoked. Smoking cessation also reduces inflammatory markers and hypercoagulability, rapidly improves the levels of high-density lipoprotein cholesterol (which protects against heart attack), and may lead to improved endothelial function. Smoking cessation reduces the development and progression of markers of subclinical atherosclerosis, with larger reductions shown in cases where the cessation period has been substantial.

In patients with existing stroke, smoking cessation reduces risk of stroke morbidity and mortality. The evidence is strongest for subarachnoid haemorrhage and less consistent for intracerebral haemorrhage. After some years of smoking cessation, the risk of stroke approaches that of those who never smoked.

Respiratory disease: Smoking cessation reduces asthma symptoms, improves treatment outcomes and asthma-specific quality of life scores among persons with asthma, and improves peak expiratory flow among persons with asthma who smoke. However, it is not sufficiently clear if smoking cessation among smokers lowers the risk of developing asthma. Indeed, the extreme smoker: non-smoker risks for respiratory disease, particularly for emphysema, match that of lung cancer (Thun et al., 2013). The benefits of cessation, once disease has begun, may have less impact on lung cancer and emphysema than on some other cancers and cardiovascular disease.

Cancers: Smoking cessation reduces risk of cancers of the lung, larynx, oral cavity and pharynx, oesophagus, pancreas, bladder, stomach, colon, rectum, liver, cervix and kidney, and acute myeloid leukemia.

### Estimates of the reduction in excess risk among former smokers

The USSGR 2014 published also a summation of the relative risks for former smokers, based on the prospective studies noted above. A comparison of excess risk, meaning RR-1 (the risk among non-smokers) for both former smokers and current smokers is informative. Based on the RRs in the USSGR report, I calculate the relative reduction in excess risk for various specific conditions (Table 5).

**Table 5.** Relative reduction in excess risk among former smokers as compared to excess risk among current smokers, by sex and age.

	Reduction in excess risk								
Disease/sex	Males				Females			
Age groups	35–54	55–64	65–74	≥75	35–54	55–64	65–74	≥75
Lung cancer	74%	80%	75%	75%	87%	78%	74%	76%
Other cancers	51%	64%	64%	61%	14%	74%	75%	71%
Coronary heart disease	71%	74%	67%	67%	69%	91%	76%	66%
Cerebrovascular disease			80%	75%			81%	86%
Aortic aneurysm, other arterial and atherosclerosis			81%	82%			78%	79%
Diabetes mellitus				40%			46%	40%
All vascular at ages 35–64	95%	66%			100%	90%		
Influenza, pneumonia, tuberculosis			61%	32%			63%	80%
Chronic obstructive pulmonary disease			75%	75%			61%	70%
All respiratory at ages 35–64	65%	79%			84%	52%		
All causes	79%	76%	72%	71%	72%	79%	72%	71%

Notes: Author calculations. The reduction in excess risk for each condition and sex is calculated as (1- [RR_f_-1)/ [RR_c_-1]), where RR_f_ and RR_c_ refer to the smoker: non-smoker relative risks (RR) for former and current smoking, respectively in the U.S. Department of Health and Human Services, 2014 (Appendix 1).

This analyses shows that, depending on age and sex (and age of cessation, which is not considered in the USSGR 2014 estimates), that versus current smokers, former smokers have about at least a three-quarters reduction in mortality from lung cancer, stroke and coronary heart disease. Reductions in excess mortality risk from chronic obstructive pulmonary disease are also about three quarters for men, but notably smaller in women. Overall, former smokers have only about a quarter of the excess risk of overall mortality versus continued smokers.

The true gain of life-years from the time of cessation could be somewhat greater than implied from Table 5. Some deaths may well reflect deaths among smokers who quit because they became ill. Large scale, well-designed epidemiological studies consider this possible ‘reverse causality’ by, for example, excluding the first few years of follow-up data (Banks et al., 2015; Jha et al., 2013; Pirie et al., 2013; Thun et al., 2013). The reductions in risks are of course strongly dependent on the age at smoking, and the calculation of RR among former smokers is likely not a robust as those for current smokers, as these prospective studies had slightly different definitions of former smoking and due to various adjustments to take into account reverse causality.

## Biological evidence of smoking hazards

Thus far, I have focused on the epidemiological evidence of the causal links of smoking to specific diseases and to overall mortality. Here, I turn briefly to the biological evidence. A common criticism of the epidemiological studies is that the exact ‘mechanism’ that causes smoking to induce lung cancer has not yet been identified. This is mostly irrelevant. As Thun et al. (2002) point out, the epidemiological evidence of association is so strong between smoking and various diseases, that further biological evidence on mechanisms is not required to establish causality. Moreover, as smoking causes a wide range of diseases, it is likely different biological mechanisms apply (for example, there may be different mechanisms at the cellular level in cancers than in cardiovascular diseases).

Doll and Peto (1981) illustrate that human population trends have distinct advantages in studying smoking as an exposure. In Canada, the US and the UK, there have been substantial earlier increases and more recent decreases in smoking-attributable mortality and from specific conditions from about 1970. During that time period, the genetic susceptibility of the relevant populations has not likely changed; say, towards reduced expression of the genetic factors that predict lung cancer or genetic factors that decrease addictiveness to nicotine. Increases and the more recent decreases in smoking account for most of the dramatic changes in smoking-attributable diseases in recent decades; genetics has likely played only a minor or no role in explaining the marked changes in tobacco-attributable mortality in recent decades.

### Biologic evidence on nicotine addiction

The nicotine in cigarettes is the central ingredient that leads to initiation and sustained smoking. Prior to about the mid-1980s, the common understanding was that tobacco use did not qualify as a drug addiction (Koop, 2003). The UK’s Medical Research Council and the US Surgeon General’s Office began to review their evidence using the logic that tobacco prevention and addiction treatment required a better understanding of the addictive properties of nicotine and cigarettes as an effective and toxic delivery system. The findings of the 1988 US Surgeon General’s report suggest that cigarettes and other forms of tobacco (such as chewed tobacco) are addicting and that nicotine is the major agent responsible for this addiction (U.S. Department of Health and Human Services, 1988). These findings have been supported by many subsequent studies and reports. In 2000, the British Royal College of Physicians concluded: “*Nicotine is an addictive drug, and the primary purpose of smoking tobacco is to deliver a dose of nicotine rapidly to receptors in the brain…Tobacco smoke inhalation is the most highly-optimized vehicle for nicotine administration…”* (Royal College of Physicians of London, 2000). In most aspects of dependence, nicotine is on par with other powerfully addictive drugs, such as heroin and cocaine.

The epidemiological evidence on smoking trends and consequences is now supported by better understanding of the neurobiological mechanisms of nicotine reinforcement and dependence. Though there are over 4,000chemicals found in cigarette smoke, there is little doubt that nicotine is the major component responsible for tobacco addiction.

Nicotine is a psychoactive drug that appears to trigger a cascade of neurobiological events in the brain and throughout the body which can, in turn, act in concert to reinforce tobacco use and affect subsequent behaviour (Markou and Henningfield, 2003). Much of the psychoactive effects of nicotine can be attributed to its rapid delivery to the brain. Absorption of cigarette smoke is accelerated and complete, with delivery of nicotine to the brain almost 10–16 seconds faster than by intravenous injection (Jarvis, 2004). Moreover, each subsequent exposure to cigarette smoke leads to the establishment and strengthening of tolerance to the adverse effects of nicotine, physiological dependence and the biologically rewarding effects of nicotine. This cascade of effects can lead to increased self-administration and progression of the dependence process. It is not known if all nicotine-induced changes in brain function, such as tripling of the levels of certain brain neurotransmitters in some brain regions (Perry and Chalkley, 1982), and alterations of brain nicotine reinforcement systems (Laviolette and van der Kooy, 2004; Mansvelder et al., 2002), are fully reversed after nicotine abstinence. It is plausible that persisting brain alterations may confer a continuing need for nicotine in some individuals (Henningfield and Slade, 1998). While all adults are susceptible to the biological effects of nicotine, it also appears plausible that early onset of use is associated with a higher risk of developing dependence. Jarvis notes that, “*in experimental models, if nicotine’s neurobiological effects are blocked pharmacologically, or if nicotine is removed from cigarette smoke, then smoking eventually ceases”*. The neurobiological effects of nicotine differ greatly from that of other licit and illicit substances. For example, for most alcohol drinkers, there is not a comparable neurobiological dependence on repeat ‘hits’ (ingestions), as there is with nicotine (Jarvis, 2004).

### Biological evidence for smoking and cancer

Proctor (2012) reviews the history of the early biological evidence that linked smoking to cancer, which I summarize. Early studies in the 1930s and early 1940s in Argentina and in Germany (published mostly in Spanish and German) examined the application of ‘tobacco juice’ to the skin and other organs in laboratory animals and showed that painting the tar of cigarette smoke on the shaved backs of mice generated tumours in the mice. The cigarette industry ridiculed these findings and funded alternatives to counter these discoveries. Next, pathologists established that smoking interfered with the small hair-like structures (cilia) in the lungs that were responsible for clearing contaminants in the lungs (Hilding, 1956). Investigations on cancer-causing chemicals in cigarette smoke such as polycyclic aromatic hydrocarbons and later benzopyrene were also underway around this time. The cigarette industry’s own research similarly identified such ingredients in their products. By the end of the 1950s, cigarette manufacturers had characterized several dozen carcinogens in cigarette smoke, including arsenic, chromium, nickel and a wide variety of polycyclic aromatic hydrocarbons.

To date, over 7,000 chemicals have been identified in cigarette smoke, including acetone (solvent and paint stripper), ammonia (powerful and poisonous gas), arsenic (potent ant poison), benzene (poisonous toxin), butane (flammable chemical in lighter fluid), cadmium (employed in batteries), carbon monoxide (poisonous gas in auto exhaust), formaldehyde (preservative for dead bodies), hydrogen cyanide (deadly ingredient in rat poison), methanol (jet engine and rocket fuel), polonium-210 (radioactive element) and toluene (poisonous industrial solvent) (IARC Working Group on the Evaluation of Carcinogenic Risk to Humans, 2004; U.S. Department of Health and Human Services, 1989; U.S. Department of Health and Human Services, 2014). Many of these have been classified independently as carcinogens by the rigorous IARC review process (IARC Working Group on the Evaluation of Carcinogenic Risk to Humans, 2004).

More recent evidence has identified the role of tobacco smoking in triggering or enabling possible mechanisms of cancer, most notably somatic mutations (meaning genetic alternations that are passed on during cell replications). Smoking also appears to alter DNA methylation, one of the main forms of epigenetic modification (meaning smoking can change how genes express into proteins, without changing the DNA itself) (Vucic et al., 2014). A recent review of the somatic mutations and DNA methylation in cancers of types for which tobacco smoking confers an elevated risk, found that smoking caused multiple mutations, most notably the misreplication of DNA by tobacco carcinogens, or indirect activation of DNA editing (Alexandrov et al., 2016). Finally, the p53 protein is responsible for a wide range of factors that suppress cancerous growth. Smoking-related malignancies have a high genome-wide burden of mutations, including in gene encoding for p53 (Gibbons et al., 2014).

By contrast, one paper suggested that ‘bad luck,’ that is random errors in DNA replication, were responsible for variation in cancer risk among 25 different types of cancer (Tomasetti and Vogelstein, 2015). This paper observed a strong correlation between the number of lifetime stem cell divisions in an organ and the lifetime organ-specific cancer risk in the US. The authors concluded that luck, far more than ‘environmental or genetic susceptibility,’ accounted for these cancers. As Blot and Tarone (2015) review, this is a misleading analysis to determine cancer causation. Any particular cancer usually requires multiple genetic changes (Peto, 2016). Both random errors and those due to genetic susceptibility or damage from environmental causes, such as smoking, increase with the total number of stem cell divisions. Hence, both types of mutations would contribute to the observed positive correlation between stem cell divisions and lifetime cancer risk. Blot and Taroneconclude, *“…the mutation rates and the totals of lifetime stem cell divisions at various organs or tissues are not likely to differ widely among different human populations, and thus even if most mutations in the majority of cancers are the result of random replication errors, the large geographic variation in cancer rates observed in most organs suggests that the percentage of cancers arising entirely by such random errors is relatively low”* (Blot and Tarone, 2015).

### Biological evidence for smoking and vascular disease and diabetes

The US Surgeon General provides detailed reviews of possible mechanisms that link smoking to vascular disease. The 2004 Surgeon General’s report provided a detailed overview of mechanisms linking smoking with cardiovascular diseases development. The report concluded that smoking (1) promotes harm to the linings of the cardiac arteries; (2) produces a substantial shift in blood based factors, clotting, and inflammation, all of which can contribute to sudden heart attack; (3) diminishes the ability of the blood to carry oxygen; (4) increases physiologic demands of the heart; and (5) causes irregular heartbeats (arrhythmias and spasm) (U.S. Department of Health and Human Services, 1990; U.S. Department of Health and Human Services, 2004; U.S. Department of Health and Human Services, 2014). Through these mechanisms, smoking results in substantial adverse alterations in the haemostatic balance of the cardiovascular system, which explains the relationships between smoking and subclinical and clinical manifestations of atherosclerosis.

The USSGR 2010 report reviewed in detail the mechanisms through which cigarette smoking causes coronary heart disease (U.S. Department of Health and Human Services, 2010b), concluding that smoking produces insulin resistance that could, in tandem with chronic inflammation, accelerate the development of damage to the arteries supplying the heart and kidney, and other organs. The 2014 USSGR expanded on the research related to mechanisms through which smoking affects cardiovascular function, focusing on how smoking affects coronary artery narrowing, blood clotting and inflammation (U.S. Department of Health and Human Services, 2014). Csordas and Bernhard (2013) thoroughly reviewed the biology of these effects of smoking and found similar conclusions. A recent examination in rat models noted that nicotine alone increases the body’s stress response, raising blood glucose levels, and thus diabetes (Duncan et al., 2019). This finding might help explain the higher prevalence of diabetes among cigarette smokers. It would also suggest similar effects on diabetes from prolonged use of nicotine in e-cigarettes.

Most of these mechanisms take less time from exposure to disease than is the case with cancer. Hence, these findings may help to explain why smoking cessation induces much faster reversibility of heart disease risk than it does for common cancers. These findings also help to explain why the relative risks for heart attack are much greater in younger than in older smokers (Table 4).

## Scientific and popular understanding of smoking risks

Despite about 40,000 studies since about 1950 on the relationship between smoking and disease, there continues to be widespread ignorance about the hazards of smoking by the public, non-experts and even some experts. For example, adults do not know that smoking is a cause of stroke based on numerous surveys, particularly in low-income countries or among less educated adults in high-income countries (Jha and Chaloupka, 1999; Jha and Chaloupka, 2000). What is surprising however, is the extent to which even informed professionals can be unaware of the hazards of smoking.

This confusion about smoking hazards in part reflects definitions of causality. Consider lung cancer, which is among the most widely-studied of the tobacco-attributable diseases. Despite the common myth, smoking is not the only cause of lung cancer. Smoking is a cause of about 90% of lung cancer deaths in high-income countries. However lung cancer occurs (albeit rarely) in non-smokers. These rare occurrences may be related to factors such as radon exposure, though it is useful to note the rate of lung cancer among non-smokers has changed little over time (Thun et al., 2008). Hence, smoking is not a *necessary* cause of disease. Some smokers do not die a premature death from their smoking, and not every smoker will develop lung cancer (or heart disease or other tobacco-attributable conditions). Hence, smoking is not a *sufficient* cause of disease. With that said, smoking is an *important cause* of lung cancer and other diseases. In epidemiological terms, when we state that smoking is an important cause of lung cancer, we are stating that, among people of the same age, smokers have a (statistically significant) increased probability of developing lung cancer in the near future than do otherwise similar non-smokers. Similarly, smoking is an important cause of vascular and respiratory disease and other diseases (U.S. Department of Health and Human Services, 1989).

Once causality is established for a particular condition, it is important to then consider the implications for public health. Here, we have to understand the relevant concept of the combination of two risks, together, as a cause of disease. For example, asbestos was used widely among shipyards in the UK that were built during WWI and WWII, prior to awareness of the hazards of asbestos on lung cancer and respiratory disease. Similar Canadian workers faced lower risks in part as the type of asbestos exposure was less carcinogenic than in the UK (Peto, 1985). Thus, large numbers of male UK shipyard workers were exposed to asbestos dust during each working day for many years. In these men, lung cancer developed at about ten times the rate of its development in otherwise similar men not exposed to asbestos dust (Barlow et al., 2017). Among these male shipyard workers, many smoked, and smoking made new lung cancer ten times more likely. Thus, shipyard workers who smoked and were exposed to asbestos dust were, by age 60 years, 100 times as likely to get lung cancer as men who had never smoked and who had not been exposed to asbestos dust (Markowitz et al., 2013).

If around 1950, we took 100 smokers who were exposed to asbestos and died of lung cancer, 90 of those 100 lung cancer deaths would not have happened if the individual had not smoked. Similarly, 90 of those 100 lung cancer deaths would not have happened if the individual had not been exposed to the particular types of asbestos dust. Thus, smoking and asbestos are each a cause of 90 of these 100 lung cancer deaths. The attributed fractions obviously add up to over 100%. Naturally, it follows that avoidance of both smoking and asbestos exposure would not avoid much more than the 90 deaths that each caused. Moreover, using the same analogy as above for asbestos, the fractions caused by genetic factors and caused by environmental factors are not mutually exclusive (Doll and Peto, 1981). A specific disease has both genetic and environmental causes.

In addition to misunderstanding of causality, two additional aspects of the divergence between the scientific evidence and popular understanding: underestimation of smoking hazards and confusion with other risks.

### Underestimation of the hazards of smoking

Widespread underestimation of the hazards of smoking arises from the long delay between uptake of smoking and the development of most diseases, as discussed earlier. Smoking is so common that many people appear to believe that something so widely used cannot be harmful. Finally, as smoking does not kill each of its users, there is always the reference to individual anecdote: many people will personally know a relative or family friend who smoked to age 85 and died peacefully in his sleep. Of course, the many mothers and fathers who died early in adult life and smoked usually do not appear often in these anecdotes.

Consider two examples of the misunderstanding of the hazards of smoking and the benefits of cessation. The first is excerpted from the notable journal *The Economist*, which is read widely by government officials and academics around the world:

*“The public-health rhetoric often implies that smoking must be daft, because it is deadly. In fact, most smokers (two-thirds or more) do not die of smoking-related disease. They gamble and win. Moreover, the years lost to smoking come from the end of life, when people are most likely to die of something else anyway. [Then US President] Bill Clinton's mother, who died of cancer at the age of 70 after smoking two packs a day for most of her life, might, as Mr. Clinton notes, have extended her life by not smoking; but she might also have extended it by eating better or exercising more, and in any case she could never have been sure.”* (The Economist, 1998).

The second is by Nobel-Prize winning economist Angus Deaton, who, in his 2013 book, *The Great Escape*, wrote:

*“Although smokers are ten to twenty times more likely to die of lung cancer than non-smokers, the vast majority of smokers do not die of the disease; the Memorial Sloan-Kettering Cancer center has an online calculator that estimates the risks. For example, a 50 year old man who has smoked a pack a day for thirty years has a one percent chance of developing lung cancer if he quits now and a two percent chance if he does not.”* (Deaton, 2013, p. 135).

The epidemiological evidence does not support either assertion. Firstly, smoking kills at least half of all those who smoke continuously and the years of life lost are substantial in middle-age. Moreover, *The Economist* quote confuses the fact that ‘eating better’ and ‘exercising more’ do little to offset the harmful effects of smoking. The confusion of big and small risks is a common mistake in media reporting of scientific research, as I take up further in the next section.

Angus Deaton substantially underestimates the actual risks of death from lung cancer. Among US men aged 50 who continue to smoke, close to 16–20%, not 2%, will be killed by lung cancer. Quitting smoking by age 50 reduces the risk of death from lung cancer by about two-thirds, not by one-half (Jha, 2009; Peto et al., 2000; U.S. Department of Health and Human Services, 2014; Pirie et al., 2013; Figure 11). Reassuringly, *The Economist* has changed its stance on tobacco completely, and now argues for much higher excise taxation and other approaches to reduce tobacco deaths (The Economist, 2017).

It is therefore unsurprising to note that among US adults surveyed randomly (Oklahoma Tobacco Research Center, 2017), there was widespread lack of awareness of the levels of risk conferred by smoking. While 82% of the adults in the US survey knew that smoking caused heart disease, emphysema, leukemia and various cancers, only 41% knew that smoking kills on average 1,200 Americans every day. Only 37% knew that more people die from smoking then from murder, AIDS, suicide drugs, car crashes and alcohol combined. Similarly, 90% of the respondents stated that smoking is highly addictive and that nicotine is the addictive drug in tobacco. High proportions of this population believed that it was not easy to quit smoking. However less than 65% of respondents knew that cigarette companies intentionally designed cigarettes with enough nicotine to create and sustain addiction. Just over 60% knew that nicotine affects the brain, making cessation quite difficult for most. This survey also noted low levels of awareness regarding the fact that low-tar and light cigarettes are as equally harmful as regular cigarettes (U.S. Department of Health and Human Services, 2014). More recently, the perception of risk of e-cigarettes as being as harmful as cigarettes has become common, and mostly likely a consequence of the considerable media attention focused on youth gateways to smoking or to addiction (McNeill et al., 2020).

### Confusion of the large hazards of smoking with the smaller hazards of most other exposures

Let’s consider one of the statements made by the British American Tobacco, a global multi-national cigarette manufacturing, in response to requests made by the US Congress in 2003 (Waxman, 2003) about their belief of the causal role of cigarette smoking:

*“…that causes of lung cancer, chronic obstructive pulmonary disease, and cardiovascular diseases are complex, and the mechanism of causation, as well as the possible role of any cigarette smoke constituent in causation, have not been scientifically established”* (Waxman, 2003).

Over the last decade, many tobacco companies have admitted that smoking causes disease, but most continue to fudge their belief in causality. Indeed many politicians have made similar arguments that minize the hazards of smoking. For argument’s sake, let’s extend the tobacco industry concerns about causality to a widely held suggestion that other ‘complex’ factors are responsible for smoking harms at the population level or in particular individuals. These other factors, based on common sense, should be closely associated with being a smoker. These might include lack of exercise, poor nutrition, alcohol drinking, peer pressure to smoke, low social status, psychology influences, and stress. Additional factors that might also be more common in smokers could include poor in-utero environment, poorer access to medical treatment, food choices, and the emerging area of science on the gut microbiome environment (Finlay and the Microbiome, 2020).

A noted UK geneticist, Sir Ronald Fisher, attacked the Doll and Hill studies of the early 1950s, suggesting that the case-control study design could not establish causality, instead hypothesizing that there was an underlying gene that resulted both in smoking and in lung cancer. Fisher was later reported to have received funding from the tobacco industry and eventually recanted some of his criticisms of Doll and Hill (Christopher, 2016).

Careful epidemiological studies have been able to examine many, but certainly not all of the first list of factors that by common sense occur in smokers. These find, for the most part, that ‘adjustment’ for such differences between smokers and non-smokers makes little difference to the observed smoker: non-smoker relative risks. What then of unmeasured variables or as yet unknown variables, as these are, by definition, bound to exist in any study? It would be expected that even undetermined factors should, for the most part, also correlate with known factors. Thus a mystery factor, either behavioural, environmental or genetic, that I will call ‘Zulu’ likely correlates closely with poor nutrition, lack of exercise as well as to smoking. It follows that, were Zulu the main true explanation for the observed smoker: non-smoker risks, adjustment for a closely correlated surrogate like lack of exercise would reduce the observed smoking relative risks. Most careful studies find, in fact, little diminution of smoker: non-smoker risks with such adjustment. This suggests that ‘residual confounding’ with unknown or unexplained risk factors does not explain most of the large observed risks for smoking. It also suggests that smoking acts independently of the other factors, even though they may be correlated.

Indeed, adjustment for smoking explains many of these other factors. Consider low social status, which is well described as a risk factor for many diseases. The UK Million Women’s study examined the social differences in hospitalization or death from ischemic heart disease. Heart disease death or hospitalization was strongly associated with lower levels of education or greater (neighborhood) deprivation, with clear dose-responses. However, smoking, alcohol consumption, physical inactivity and body mass index accounted for most of the risk factors, and of these four factors, smoking accounted for the biggest share (Floud et al., 2016; Jha et al., 2019).

Any particular disease can have in fact two (or more) causes. Therefore, obesity and smoking may both contribute to heart disease and avoidance of either might have prevented a particular heart attack. However, from a population health perspective, what is useful is to understand the comparative risks for each and to what extent are these risks avoidable.

I now review some of these major identified ‘other factors’ beginning first with obesity (presumed to be both ‘physiological’ and ‘based on lifestyle choices’ in tobacco industry parlance), then turning to alcohol, and finally genetics and environment. I selected these factors as the WHO has identified these as major risk factors for adult health (World Health Organization, 2016) and because clinicians and the public often turn to genetic differences to explain disease occurrence. Moreover, smoking and drinking, and to a lesser extent obesity are strongly correlated in healthy as well as in diseased individuals (Thun et al., 1997).

#### Comparison with obesity

**Figure 12.**
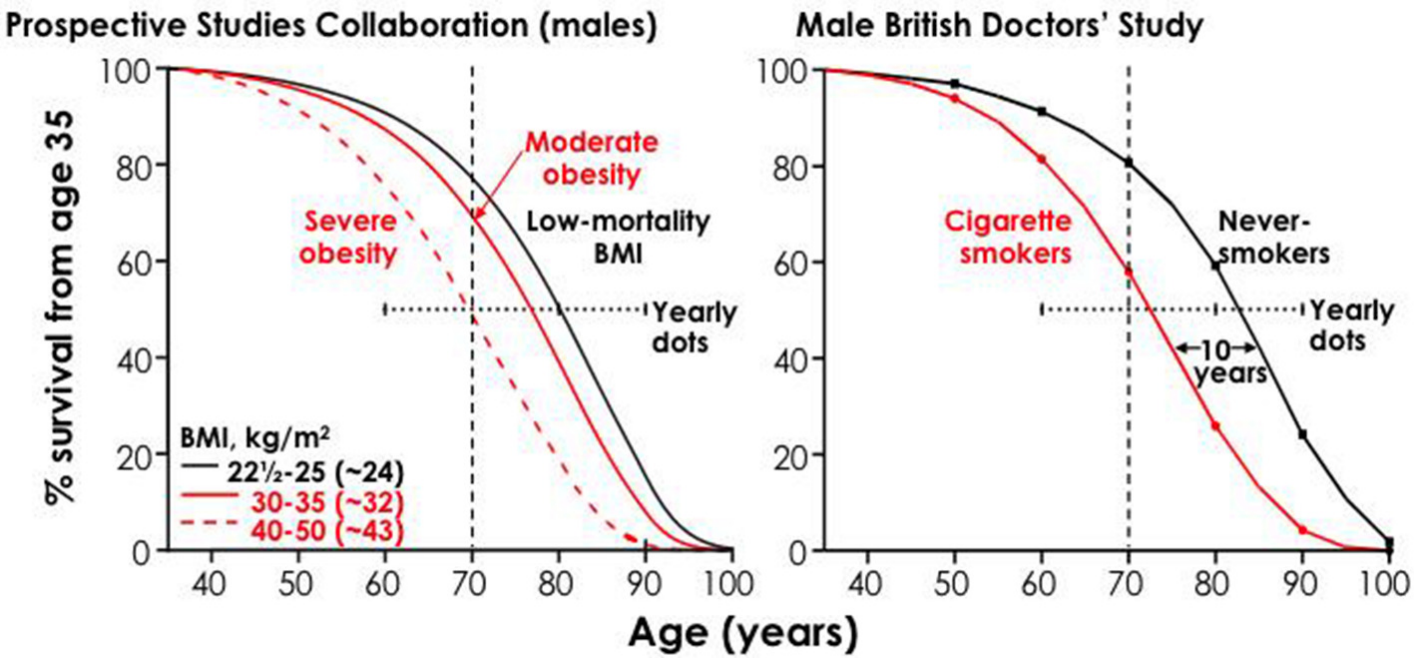
Life expectancy: loss of 3 years with moderate obesity and 10 years with smoking. Note: 2 kg/m^2^ extra BMI (if overweight) or 10% smoking prevalence shortens life by ~1 year. Adapted from Peto et al. (2010), p. 856.

Smoking risks are substantially greater than those of obesity for adults in high-income countries. Obesity (at its most extreme called severe obesity) is defined as excess body fat, and measured by body-mass index (BMI; or weight divided by height squared). Higher BMI causes a loss of premature life (Finucane et al., 2011). The loss of a decade of life requires a BMI of around 43, which is well above the averages seen in any country including the most obese populations of the US. More moderate levels of obesity, meaning of BMI of around 32, contibute to an average of three years of life loss. Hence, at the population level, a 2 kg per meter extra BMI if overweight or a 10% higher smoking prevalence both reduce life expectancy by one year (Figure 12; Peto et al., 2010).

These individual risks also translate to the overall life expectancy gaps at the population level. About 15% of American smoke cigarettes, and smoking accounts, on average, for 10 years of life lost. Thus, smoking is reducing American life expectancy by about 1.5 years of life (i.e. 15% X 10 years). The mean BMI in the US is 28 and about 30% of the American population is modestly obese. If modest obesity accounts for 3 years of life lost, then obesity is reducing American life expectancy by 0.9 years (i.e. 30% X 3 years). Hence, smoking causes more lost life than does obesity in the US. Moreover, the main mechanisms for increased mortality from higher BMI from vascular disease are due to hypertension, ‘bad’ lipids and development of diabetes. Most of these factors driven by higher BMI are very treatable even without reducing weight (Yusuf et al., 2004; Yusuf et al., 2020). By contrast, the only plausible way to reduce smoking risks is by quitting (Jha et al., 2013).

#### Comparison with alcohol

Heavy alcohol use increases the death rate from some conditions (most notably, road traffic and other injuries, suicide, poisoning, liver cirrhosis, certain cancers and perhaps haemorrhagic stroke) (Thun et al., 1997); and recent studies have documented hazards from drinking for ischemic stroke and no protective effect for ischemic heart disease (Millwood et al., 2019). Thus, the overall survival of otherwise comparable drinkers and non-drinkers in Canada, the US and the UK are similar (the slightly greater survival in drinkers shown in earlier studies is mostly likely due to ‘reverse causality’ whereby sicker people give up drinking). This balance of risks is distinct from the extreme binge-drinking patterns of vodka among Russian men, in whom loss of life from such binge-drinking exceeds that of smoking (Zaridze et al., 2009; Zaridze et al., 2014). However, the hazards of smoking are observed in both drinkers and non-drinkers (Table 6).

**Table 6.** Estimated probability of death (%) from any cause between ages 35 and 69 in the US population, by combination of alcohol drinking and smoking, in 1990.

Sex and alcohol drinking status	Smokers	Non-smokers
MEN		
Drinkers	43	22
Non-drinkers	46	26
WOMEN		
Drinkers	28	14
Non-drinkers	30	17

Adapted from *Alcohol consumption and mortality among middle-aged and elderly US adults*, by Thun et al. (1997), *New England Journal of Medicine, 337*(24), p. 1712.

Even epidemiologists often underestimate the extreme hazards of tobacco. Consider the following comparisons. Firstly, among 1,000 male smokers aged 20, at least 500 (and perhaps up to 670), will die from smoking throughout their lifetimes. Of these, at least 250 will die from smoking in middle-age (meaning before 70 years). By contrast, only 20 would die from road accidents or violence and 30 from all alcohol-related conditions (Jha and Chaloupka, 2000).

#### Comparison with genetics

To return to the idea of causation; a common statement made by people (including doctors) is that ‘genetics was responsible’ for a particular person’s heart attack or lung cancer, even though they may have also smoked. At the population level, both genetics and smoking could for example, play roles in causing either disease. The population-based evidence should also guide the magnitude of the risk. For example, no single genetic factor has been identified that explains a significant proportion of heart attacks, although the combination of all tested factors suggest just over a quarter of heritability of heart disease (McPherson and Tybjaerg-Hansen, 2016).

However, smoking alone accounts for at least a quarter of the deaths from heart attack in the US (U.S. Department of Health and Human Services, 2014). Moreover, the largest identified single genetic factor (the relative risk from a specific lipid factor (lipoprotein (a), Loci *SLC22A3-LPAL2-LPA* SNP, *rs2048327*) carries a relative risk of 1.4, which is comparable to smoking 3–4 cigarettes a day. Similarly, a range of genetic factors has recently been suggested to modestly predict lung cancer risk in smokers and non-smokers (Timofeeva et al., 2012). These genetic factors do not negate the importance of smoking in explaining lung cancer risks.

#### Comparison to environmental pollutants and other exposures

Doll and Peto (1981) conducted an exhaustive review of the possible causes of cancer in the US in the late 1970s. Their focus was on the widely held belief that ‘pollutants’ in the environment were a major cause of cancer. They documented the available epidemiological evidence on the environmental exposures most commonly believed to be linked to cancer, such as pesticides, food additives, industrial products, ultraviolet light, and pollution. (They did not specifically study ambient air pollution). They concluded that tobacco smoking accounted for more of the proportions of cancer deaths than did a reasonable summation of all of the known risk factors, including nearly every known pollutant. Table 7 shows their summary results.

**Table 7.** Proportions of cancer deaths in the United States in the 1970s attributed to various different factors, as defined by Doll and Peto (1981).

Factor or class of factors	Percent of all cancer deaths	
	Best estimate	Range of acceptable estimates
Tobacco	30	25–40
Alcohol	3	2–4
Food additives	<1	−5–2
Reproductive and sexual behaviour	7	1–13
Occupation	4	2–8
Pollution	2	<1–5
Industrial products	<1	<1–2
Medicines and medical procedures	1	0.5–3
Geophysical factors (mostly Ultraviolet light)	3	2–4
Infection	10?	1-?
Diet	35	10–70
Unknown	?	?

Adapted from *The causes of cancer: Quantitative estimates of avoidable risks of cancer in the United States today*, Doll and Peto (1981), *Journal of the National Cancer Institute, 66*(6), p. 1192–1308.

Doll and Peto also pointed out that, excluding smoking-attributable cancers, there was no ‘epidemic’ of cancer, as was commonly believed at that time. In fact cancer death rates except those attributable to smoking were falling modestly. Finally, they pointed out that smoking causes more death from causes other than cancer than it does from cancer itself.

## Future research directions and conclusions

In this review, I have considered the cause, nature and extent of tobacco related diseases in high-income countries between 1960 and 2020. I have drawn on existing epidemiological evidence, with careful re-interpretation of risks in individuals and rates among populations. It follows naturally to ask what additional epidemiological or biological evidence is needed on the consequences of smoking in high-income countries. A full treatise on research priorities is beyond the scope of this review, but a few priorities should be considered. First, the rapidly changing prevalence of smoking, including increases in cigarette cessation require ongoing studies to document the benefits of quitting, particularly on various diseases and at different ages (U.S. Department of Health and Human Services, 2020). Second, the emergence of e-cigarettes demands further documentation of the long-term risks (and possible benefits) of their use among adults and by adolescents. The current state of evidence is mostly insufficient for conclusions on the net effects of e-cigarettes on population health. Third, research to better understand how risks are perceived and internalized by individuals and governments is needed to ensure that epidemiological studies on the consequences of smoking are incorporated into decision-making.

Based on the existing epidemiological and biological evidence, I provide four overall conclusions.

Firstly, in much of North America and Western Europe, the biggest cause of premature death, defined as death before 70 years, is the smoking of manufactured cigarettes. Smoking as an important cause of many diseases in many populations has been recognized widely in the scientific literature for the last five decades. However, three surprising features of health hazards of smoking have been established reliably only in the last decade. The first feature is that risk of developing disease among smokers is big. The second feature is that for smokers to develop these big risks, they need to start smoking early in adult life and to continue smoking. If smokers don’t start early in life, their risks are substantially smaller. Third, if smokers stop smoking before they develop some serious disease, then their risks are substantially reduced. However, most smokers whom start early in adult life and who continue to smoke are eventually killed by their tobacco use. This is because in every year during middle age, the death rates among smokers are about three-fold higher than that of similar non-smokers (taking into account differences between smokers and non-smokers in heavy alcohol use, obesity patterns or different social status). So up to two-thirds of the mortality among smokers would not be happening if they had the non-smoker death rates. Most of this excess risk arises from diseases that are caused by smoking. This includes disease such as lung cancer, emphysema, heart attack, stroke, cancer of the upper aerodigestive areas, bladder cancer and various other conditions. Thus this excess risk is a cause and effect relationship.

Secondly, from 1960 to 2020, smoking has likely killed 29.5 million Americans, 9.3 million UK residents, and 2.6 million Canadians, or a total of 41.3 million adults. This constitutes a crude ratio of one death per million cigarettes smoked in the US and Canada, but slightly more than one death per million cigarettes smoked in the UK.

Third, cessation, particularly before age 40 avoids nearly all the excess risk of continued smoking. Cessation at any age is effective, restoring substantial years of like lost versus continued smoking.

Finally, there continues to be widespread, serious underestimation of the hazards of smoking by the public, non-experts and even some experts.

## Appendix 1

**Appendix 1—table 1.** Relative risks for various diseases by smoking status, adults 35 or older in the United States.

	Current smokers (years of age)				Former smokers (years of age)			
	35-54	55-64	65-74	≥75	35-54	55-64	65-74	≥75
MALES								
Lung Cancer	14.33	19.03	28.29	22.51	4.4	4.57	7.79	6.46
Other Cancers	1.74	1.86	2.35	2.18	1.36	1.31	1.49	1.46
Coronary heart disease	3.88	2.99	2.76	1.98	1.83	1.52	1.58	1.32
Other heart disease			2.22	1.66			1.32	1.15
Cerebrovascular disease			2.17	1.48			1.23	1.12
Other vascular diseases			7.25	4.93			2.2	1.72
Diabetes mellitus			1.5	1			1.53	1.06
Other cardiovascular diseases	2.4	2.51			1.07	1.51		
Influenza, pneumonia, tuberculosis			2.58	1.62			1.62	1.42
Chronic obstructive pulmonary disease			29.69	23.01			8.13	6.55
Influenza, pneumonia, tuberculosis, chronic obstructive pulmonary disease	4.47	15.17			2.22	3.98		
All Causes	2.55	2.97	3.02	2.4	1.33	1.47	1.57	1.41
								
FEMALES								
Lung Cancer	13.3	18.95	23.65	23.08	2.64	5	6.8	6.38
Other Cancers	1.28	2.08	2.06	1.93	1.24	1.28	1.26	1.27
Coronary heart disease	4.98	3.25	3.29	2.25	2.23	1.21	1.56	1.42
Other heart disease			1.85	1.75			1.29	1.32
Cerebrovascular disease			2.27	1.7			1.24	1.1
Other vascular diseases			6.81	5.77			2.26	2.02
Diabetes mellitus			1.54	1.1			1.29	1.06
Other cardiovascular diseases	2.44	1.98			1	1.1		
Influenza, pneumonia, tuberculosis			1.75	2.06			1.28	1.21
Chronic obstructive pulmonary disease			38.89	20.96			15.72	7.06
Influenza, pneumonia, tuberculosis, chronic obstructive pulmonary disease	6.43	9			1.85	4.84		
All Causes	1.79	2.63	2.87	2.47	1.22	1.34	1.53	1.43

*Source:* USSGR Report, 2014. Analyses of Cancer Prevention Study II (CPS-II) and updated analyses of the pooled contemporary cohort population described in Thun et al. (2013) provided to the Centers for Disease Control and Prevention, National Center for Chronic Disease Prevention and Health Promotion, Office on Smoking and Health. See Table 12.3 of the USSGR Report for important details on each condition.

**Appendix 1—table 2.** Estimates of smoking-attributable deaths in US, UK, and Canada from 1950 to 2015.

	US				UK				Canada			
Year	No of cigarettes sold nationally	Deaths from smoking at all ages	Cigs/death no lag	Cigs/death 20 year lag	No of cigarettes sold nationally	Deaths from smoking at all ages	Cigs/death no lag	Cigs/death 20 year lag	No of cigarettes sold nationally	Deaths from smoking at all ages	Cigs/death no lag	Cigs/death 20 year lag
1950	3,60,19,90,00,000	*82,350*	*43,74,001*		85,14,50,00,000	*78,000*	*10,91,603*		17,17,20,00,000	***4,350***	***39,47,586***	
1951	3,79,72,50,00,000	*93,660*	*40,54,292*		89,33,50,00,000	*82,800*	*10,78,925*		15,67,20,00,000	***5,240***	***29,90,840***	
1952	3,94,10,90,00,000	*1,04,970*	*37,54,492*		90,40,00,00,000	*87,600*	*10,31,963*		17,84,40,00,000	***6,130***	***29,10,930***	
1953	3,86,82,60,00,000	*1,16,280*	*33,26,677*		92,69,50,00,000	*92,400*	*10,03,193*		21,00,00,00,000	***7,020***	***29,91,453***	
1954	3,68,72,50,00,000	*1,27,590*	*28,89,921*		95,23,00,00,000	*97,200*	*9,79,733*		22,11,60,00,000	***7,910***	***27,95,954***	
1955	3,82,06,10,00,000	1,38,900	27,50,619		98,67,00,00,000	1,02,000	9,67,353		24,57,60,00,000	8,800	27,92,727	
1956	3,93,15,40,00,000	1,50,210	26,17,362		99,56,00,00,000	1,06,800	9,32,210		27,00,00,00,000	9,690	27,86,378	
1957	4,09,43,60,00,000	1,61,520	25,34,894		1,02,25,00,00,000	1,11,600	9,16,219		30,14,40,00,000	10,580	28,49,149	
1958	4,36,35,40,00,000	1,72,830	25,24,758		1,04,02,00,00,000	1,16,400	8,93,643		32,40,40,00,000	11,470	28,25,109	
1959	4,53,68,10,00,000	1,84,140	24,63,783		1,06,60,00,00,000	1,21,200	8,79,538		33,82,20,00,000	12,360	27,36,408	
1960	4,70,13,60,00,000	1,95,450	24,05,403		1,10,90,00,00,000	1,26,000	8,80,159		34,28,90,00,000	13,250	25,87,849	
1961	4,88,11,90,00,000	2,06,760	23,60,800		1,13,40,00,00,000	1,30,800	8,66,972		36,69,90,00,000	14,140	25,95,403	
1962	4,94,46,30,00,000	2,18,070	22,67,451		1,09,90,00,00,000	1,35,600	8,10,472		38,68,30,00,000	15,030	25,73,719	
1963	5,09,58,80,00,000	2,29,380	22,21,589		1,15,20,00,00,000	1,40,400	8,20,513		39,87,70,00,000	15,920	25,04,837	
1964	4,97,44,70,00,000	2,40,690	20,66,754		1,14,40,00,00,000	1,45,200	7,87,879		40,63,90,00,000	16,810	24,17,549	
1965	5,11,46,40,00,000	2,52,000	20,29,619		1,12,00,00,00,000	1,50,000	7,46,667		43,01,30,00,000	17,700	24,30,113	
1966	5,22,53,30,00,000	2,64,800	19,73,312		1,17,60,00,00,000	1,53,500	7,66,124		46,27,60,00,000	18,950	24,42,005	
1967	5,27,80,00,00,000	2,77,600	19,01,297		1,19,10,00,00,000	1,57,000	7,58,599		46,86,40,00,000	20,200	23,20,000	
1968	5,23,00,80,00,000	2,90,400	18,00,992		1,21,80,00,00,000	1,60,500	7,58,879		46,26,90,00,000	21,450	21,57,063	
1969	5,10,53,10,00,000	3,03,200	16,83,809		1,24,90,00,00,000	1,64,000	7,61,585		46,58,20,00,000	22,700	20,52,070	
1970	5,32,76,90,00,000	3,16,000	16,85,978		1,27,90,00,00,000	1,67,500	7,63,582		49,82,30,00,000	23,950	20,80,292	
1971	5,28,85,80,00,000	3,28,800	16,08,449		1,22,40,00,00,000	1,71,000	7,15,789		50,86,40,00,000	25,200	20,18,413	
1972	5,51,01,70,00,000	3,41,600	16,13,047		1,30,50,00,00,000	1,74,500	7,47,851		53,29,10,00,000	26,450	20,14,783	
1973	5,90,30,00,00,000	3,54,400	16,65,632		1,37,40,00,00,000	1,78,000	7,71,910		54,86,30,00,000	27,700	19,80,614	
1974	5,99,00,00,00,000	3,67,200	16,31,264		1,37,00,00,00,000	1,81,500	7,54,821		57,12,28,01,000	28,950	19,73,154	
1975	6,07,20,00,00,000	3,80,000	15,97,895	10,05,424	1,32,60,00,00,000	1,85,000	7,16,757	5,33,351	57,75,57,95,000	30,200	19,12,444	8,13,775
1976	6,13,50,00,00,000	3,92,400	15,63,456	10,01,922	1,30,60,00,00,000	1,84,900	7,06,328	5,38,453	60,74,48,85,000	31,380	19,35,783	8,60,421
1977	6,17,00,00,00,000	4,04,800	15,24,209	10,11,453	1,25,90,00,00,000	1,84,800	6,81,277	5,53,301	61,78,65,71,000	32,560	18,97,622	9,25,799
1978	6,16,00,00,00,000	4,17,200	14,76,510	10,45,911	1,25,20,00,00,000	1,84,700	6,77,856	5,63,184	61,61,00,13,000	33,740	18,26,023	9,60,403
1979	6,21,50,00,00,000	4,29,600	14,46,695	10,56,054	1,24,30,00,00,000	1,84,600	6,73,348	5,77,465	63,86,56,38,000	34,920	18,28,913	9,68,557
1980	6,31,50,00,00,000	4,42,000	14,28,733	10,63,656	1,21,50,00,00,000	1,84,500	6,58,537	6,01,084	64,34,33,00,000	36,100	17,82,363	9,49,834
1981	6,40,00,00,00,000	4,54,400	14,08,451	10,74,206	1,10,30,00,00,000	1,84,400	5,98,156	6,14,967	66,37,56,00,000	37,280	17,80,461	9,84,415
1982	6,34,00,00,00,000	4,66,800	13,58,183	10,59,261	1,02,00,00,00,000	1,84,300	5,53,445	5,96,310	66,15,74,00,000	38,460	17,20,161	10,05,798
1983	6,00,00,00,00,000	4,79,200	12,52,087	10,63,414	1,01,60,00,00,000	1,84,200	5,51,574	6,25,407	62,78,51,03,500	39,640	15,83,883	10,05,979
1984	6,00,40,00,00,000	4,91,600	12,21,318	10,11,894	99,00,00,00,000	1,84,100	5,37,751	6,21,401	62,13,39,14,500	40,820	15,22,144	9,95,566
1985	5,94,00,00,00,000	5,04,000	11,78,571	10,14,810	97,75,00,00,000	1,84,000	5,31,250	6,08,696	59,30,44,61,105	42,000	14,12,011	10,24,119
1986	5,83,80,00,00,000	5,14,900	11,33,812	10,14,824	95,00,00,00,000	1,82,000	5,21,978	6,46,154	55,76,23,21,860	43,100	12,93,789	10,73,689
1987	5,75,00,00,00,000	5,25,800	10,93,572	10,03,804	96,00,00,00,000	1,80,000	5,33,333	6,61,667	52,87,70,31,890	44,200	11,96,313	10,60,271
1988	5,62,50,00,00,000	5,36,700	10,48,072	9,74,489	97,00,00,00,000	1,78,000	5,44,944	6,84,270	51,33,89,17,800	45,300	11,33,309	10,21,391
1989	5,40,00,00,00,000	5,47,600	9,86,121	9,32,306	98,00,00,00,000	1,76,000	5,56,818	7,09,659	47,76,46,41,600	46,400	10,29,410	10,03,922
1990	5,25,00,00,00,000	5,58,500	9,40,018	9,53,928	1,02,50,00,00,000	1,74,000	5,89,080	7,35,057	46,44,08,01,080	47,500	9,77,701	10,48,905
1991	5,10,00,00,00,000	5,69,400	8,95,680	9,28,799	97,90,00,00,000	1,72,000	5,69,186	7,11,628	39,13,45,12,000	48,600	8,05,237	10,46,584
1992	5,00,00,00,00,000	5,80,300	8,61,623	9,49,538	92,80,00,00,000	1,70,000	5,45,882	7,67,647	35,20,01,50,365	49,700	7,08,253	10,72,254
1993	4,85,00,00,00,000	5,91,200	8,20,365	9,98,478	88,90,00,00,000	1,68,000	5,29,167	8,17,857	30,34,98,69,580	50,800	5,97,438	10,79,980
1994	4,86,00,00,00,000	6,02,100	8,07,175	9,94,851	88,30,00,00,000	1,66,000	5,31,928	8,25,301	45,93,47,44,885	51,900	8,85,063	11,00,632
1995	4,87,00,00,00,000	6,13,000	7,94,454	9,90,538	88,00,00,00,000	1,64,000	5,36,585	8,08,537	45,70,99,99,540	53,000	8,62,453	10,89,732
1996	4,87,00,00,00,000	6,12,400	7,95,232	10,01,796	87,20,00,00,000	1,60,700	5,42,626	8,12,694	47,33,83,59,350	53,200	8,89,819	11,41,821
1997	4,80,00,00,00,000	6,11,800	7,84,570	10,08,500	84,00,00,00,000	1,57,400	5,33,672	7,99,873	45,64,03,20,710	53,400	8,54,688	11,57,052
1998	4,65,00,00,00,000	6,11,200	7,60,798	10,07,853	84,00,00,00,000	1,54,100	5,45,101	8,12,459	45,55,66,36,420	53,600	8,49,937	11,49,441
1999	4,35,00,00,00,000	6,10,600	7,12,414	10,17,851	84,00,00,00,000	1,50,800	5,57,029	8,24,271	44,87,29,65,600	53,800	8,34,070	11,87,094
2000	4,30,00,00,00,000	6,10,000	7,04,918	10,35,246	81,50,00,00,000	1,47,500	5,52,542	8,23,729	43,36,81,94,700	54,000	8,03,115	11,91,543
2001	4,25,00,00,00,000	6,09,400	6,97,407	10,50,213	79,00,00,00,000	1,44,200	5,47,850	7,64,910	42,08,73,29,210	54,200	7,76,519	12,24,642
2002	4,15,00,00,00,000	6,08,800	6,81,669	10,41,393	76,00,00,00,000	1,40,900	5,39,390	7,23,918	37,62,74,91,460	54,400	6,91,682	12,16,129
2003	4,00,00,00,00,000	6,08,200	6,57,678	9,86,518	74,00,00,00,000	1,37,600	5,37,791	7,38,372	35,81,79,32,599	54,600	6,56,006	11,49,910
2004	3,88,00,00,00,000	6,07,600	6,38,578	9,88,150	72,00,00,00,000	1,34,300	5,36,113	7,37,156	34,57,89,18,606	54,800	6,31,002	11,33,831
2005	3,76,00,00,00,000	6,07,000	6,19,440	9,78,583	70,00,00,00,000	1,31,000	5,34,351	7,46,183	32,97,06,21,643	55,000	5,99,466	10,78,263
2006	3,80,72,63,50,521	6,01,800	6,32,646	9,70,090	67,50,00,00,000	1,41,700	4,76,359	6,70,430	30,21,71,91,194	55,300	5,46,423	10,08,360
2007	3,61,66,52,82,882	5,96,600	6,06,211	9,63,795	64,50,00,00,000	1,39,400	4,62,697	6,88,666	28,70,47,13,382	55,400	5,18,136	9,54,459
2008	3,46,41,92,70,754	5,91,400	5,85,761	9,51,133	61,50,00,00,000	1,37,100	4,48,578	7,07,513	27,55,93,82,153	55,500	4,96,565	9,25,026
2009	3,18,02,91,35,770	5,86,200	5,42,527	9,21,187	58,50,00,00,000	1,34,800	4,33,976	7,27,003	28,62,75,07,225	55,600	5,14,883	8,59,076
2010	3,00,48,93,90,443	5,81,000	5,17,193	9,03,614	52,50,00,00,000	1,32,500	3,96,226	7,73,585	31,65,36,15,884	55,700	5,68,288	8,33,767
2011	2,92,76,91,35,560	5,75,800	5,08,456	8,85,724	50,30,00,00,000	1,30,200	3,86,329	7,51,920	31,17,80,12,116	55,800	5,58,746	7,01,335
2012	2,87,48,65,11,382	5,70,600	5,03,832	8,76,271	46,00,00,00,000	1,27,900	3,59,656	7,25,567	31,34,74,37,105	55,900	5,60,777	6,29,699
2013	2,73,78,73,72,153	5,65,400	4,84,237	8,57,800	41,60,00,00,000	1,25,600	3,31,210	7,07,803	31,46,78,96,967	56,000	5,61,927	5,41,962
2014	***2,62,68,12,73,103***	***5,60,200***	***4,68,906***	***8,67,547***	***39,70,00,00,000***	***1,23,300***	***3,21,979***	***7,16,139***	***29,47,82,34,315***	***56,000***	***5,26,397***	***8,20,263***
2015	***2,62,68,12,73,103***	***5,55,000***	***4,73,300***	***8,77,477***	***39,70,00,00,000***	***1,21,000***	***3,28,099***	***7,27,273***	***29,47,82,34,315***	***56,000***	***5,26,397***	***8,16,250***
	**US**				**UK**				**CANADA**			
**TOTALS since 1950**	**3,12,38,43,79,95,671**	**2,79,01,300**			**63,06,95,50,00,000**	**98,63,000**			**27,73,85,14,67,659**	**23,67,750**		
**Average cigarettes per death with no lag**			**12,95,403**				**6,23,238**				**14,72,021**	
**Average cigarettes per death with 20 year lag**			**9,83,910**				**7,01,972**				**9,95,413**	

*Note.* Author’s calculations. Tobacco sales to deaths are shown in the column entitled ‘Deaths from smoking at all ages’. For particular years, some data were missing. In this case, I interpolated these data or did forward projections based on earlier years. These more uncertain numbers are shown in italics.
